# Phosphorylation of ezrin on Thr567 is required for the synergistic activation of cell spreading by EPAC1 and protein kinase A in HEK293T cells

**DOI:** 10.1016/j.bbamcr.2015.04.009

**Published:** 2015-07

**Authors:** Euan Parnell, Andreas Koschinski, Manuela Zaccolo, Ryan T. Cameron, George S. Baillie, Gemma L. Baillie, Alison Porter, Stuart P. McElroy, Stephen J. Yarwood

**Affiliations:** a Institute of Molecular, Cellular and Systems Biology, College of Medical Veterinary and Life Sciences, University of Glasgow, Glasgow G12 8QQ, UK; b Department of Physiology, Anatomy and Genetics, University of Oxford, Oxford OX1 3QX, UK; c Institute of Cardiovascular and Medical Sciences, College of Medical Veterinary and Life Sciences, University of Glasgow, Glasgow G12 8QQ, UK; d European Screening Centre, BioCity Scotland, Newhouse ML1 5UH, UK

**Keywords:** EPAC, exchange protein directly activated by cAMP, ROCK, RhoA activated Protein Kinase, PKA, protein kinase A, HUVEC, human umbilical vein endothelial cells, GEF, guanine exchange factor, ERM, ezrin–radixin–moesin family, FERM, 4.1 ezrin–radixin–moesin, F/R, forskolin/rolipram, 007, 8-pCPT-2′-O-Me-cAMP, F-Actin, filamentous actin, Cyclic AMP, EPAC1, Cytoskeleton, Cell morphology

## Abstract

Recent studies have demonstrated that the actin binding protein, ezrin, and the cAMP-sensor, EPAC1, cooperate to induce cell spreading in response to elevations in intracellular cAMP. To investigate the mechanisms underlying these effects we generated a model of EPAC1-dependent cell spreading based on the stable transfection of EPAC1 into HEK293T (HEK293T–EPAC1) cells. We found that direct activation of EPAC1 with the EPAC-selective analogue, 8-pCPT-2′-O-Me-cAMP (007), promoted cell spreading in these cells. In addition, co-activation of EPAC1 and PKA, with a combination of the adenylate cyclase activator, forskolin, and the cAMP phosphodiesterase inhibitor, rolipram, was found to synergistically enhance cell spreading, in association with cortical actin bundling and mobilisation of ezrin to the plasma membrane. PKA activation was also associated with phosphorylation of ezrin on Thr567, as detected by an electrophoretic band mobility shift during SDS-PAGE. Inhibition of PKA activity blocked ezrin phosphorylation and reduced the cell spreading response to cAMP elevation to levels induced by EPAC1-activation alone. Transfection of HEK293T–EPAC1 cells with inhibitory ezrin mutants lacking the key PKA phosphorylation site, ezrin-Thr567Ala, or the ability to associate with actin, ezrin-Arg579Ala, promoted cell arborisation and blocked the ability of EPAC1 and PKA to further promote cell spreading. The PKA phospho-mimetic mutants of ezrin, ezrin-Thr567Asp had no effect on EPAC1-driven cell spreading. Our results indicate that association of ezrin with the actin cytoskeleton and phosphorylation on Thr567 are required, but not sufficient, for PKA and EPAC1 to synergistically promote cell spreading following elevations in intracellular cAMP.

## Highlights

• EPAC1 is absolutely required for cAMP-induced cell spreading in HEK293T cells.
• EPAC1 facilitates further PKA-dependent cell spreading in HEK293T cells.
• PKA-dependent cell spreading is associated with cortical actin bundling.
• Ezrin is required for EPAC1- and PKA-dependent cell spreading.
• Phosphorylated ezrin (pThr567) stabilises cortical actin and prevents membrane projections.

## Graphical abstract

**Figure:**
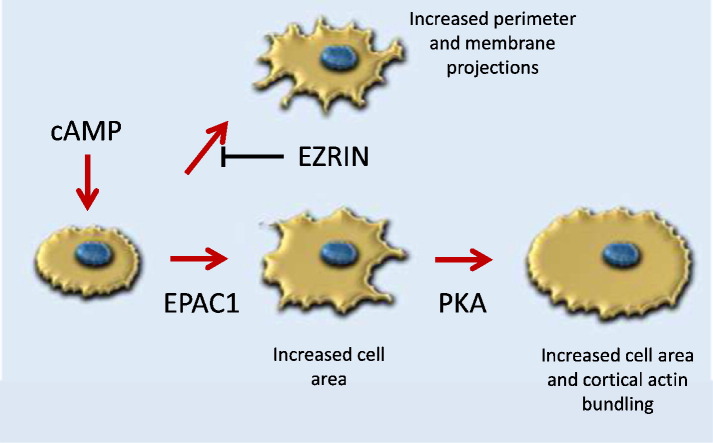

## 1 Introduction

Cyclic adenosine monophosphate (cAMP) is a ubiquitous second messenger that is involved in regulating many aspects of cell function, including cell differentiation [1], secretion [2], cell morphology [3], inflammatory pathways [4], contractility [5] and synapse formation [6]. Elevations in intracellular cAMP occur in response to activation of Gs-coupled G-protein coupled receptors (GPCRs). GPCRs, in turn, activate adenylate cyclase (AC), which catalyses the conversion of ATP into cAMP. This process is terminated through the action of the cAMP phosphodiesterase (PDE) family, which hydrolyses cAMP to 5′-AMP [7].

Elevated cAMP levels promote the activation of a select range of intracellular effectors, including exchange protein activated by cAMP (EPAC) [8,9] and protein kinase A (PKA) [10], which both contain cyclic nucleotide binding domains (CNBDs) [11]. In the case of PKA, cAMP binding to regulatory CNBDs promotes their dissociation from the catalytic subunit, thereby facilitating the phosphorylation of a plethora of downstream signalling proteins [12]. EPACs are also activated by cAMP binding, although their mechanism of activation and catalytic activity are strikingly different from PKA [8,9]. Indeed, the regulatory CNBD and catalytic regions of the two principle EPAC isoforms, EPAC1 and EPAC2, are contained within a single gene product, and regulation is imparted by an interaction between an N-terminal regulatory domain and C-terminal catalytic domain [13]. Moreover, active EPAC acts as a guanine nucleotide exchange factor (GEF) towards the small GTPases, Rap1 and Rap2 [14], as opposed to the kinase activity of PKA [10].

A large body of evidence points toward a role for EPAC1–Rap1 signalling in governing cAMP-regulated cell shape, spreading and morphology [4,15]. Furthermore, EPAC1/Rap1 signalling has been linked to the promotion of barrier protective functions in the vascular endothelium, through the regulation of adhesive and cohesive pathways linked by the dynamic cytoskeleton [16,17]. These effects are largely attributed to the re-localisation of vascular endothelial-specific VE-cadherin [18] and the regulation of cytoskeletal elements [19,20]. In particular, cytoskeletal reorganisation occurs [20,21] through Rap1-mediated regulation of the Rho GTPase family of cytoskeletal regulators [18,22,23]. However, many of these EPAC1-regulated pathways synergise with PKA for maximal effect, which may be dependent of the involvement of the ezrin–radixin–moesin (ERM) protein family member, ezrin [3].

ERM proteins are a homologous group of actin binding proteins with a characteristic N-terminal 4.1-ezrin–radixin–moesin (FERM) domain [24], which control a wide range of cellular processes through their role as scaffold between the actin cytoskeleton and the plasma membrane lipid component, phosphoinositol-4,5-bisphosphate (PIP2) [25]. The importance of ezrin in EPAC1/Rap1-mediated cell spreading has been demonstrated using siRNA-mediated knockdown of ezrin, which severely limits cell spreading in response to elevations in intracellular cAMP [3]. The activity of ezrin appears to be modulated by multiple proteins kinases, which act through phosphorylation of Ser66 [26], Tyr81 [27], Tyr145, Tyr353 [28] and Tyr477 [29]. However, it is Thr567 phosphorylation that has been identified as being key in relieving an auto-inhibitory head to tail conformation within ezrin, thereby facilitating activation [25,30]. Interestingly, phosphorylation of ezrin on Thr 567 has been reported to occur in response to cAMP elevation, through activation of PKA [31]. In this respect, ezrin may therefore serve to coordinate the actions of EPAC1 and PKA towards the regulation of cell spreading and morphological change. Here we investigate the role of ezrin in controlling the morphological response of cells to PKA and EPAC1 activation in a cell model of EPAC1-dependent cell spreading.

## 2 Materials and methods

### 2.1 Reagents

Forskolin and Y27642 were purchased from Merck, UK. 8(4-chlorophenylthio)-2′-O-methyl-cAMP (007) was purchased from Biolog Life Sciences (Bremen, Germany). H-89 was from Sigma-Aldrich and 4′, 6-diamidino-2-phenylindole (DAPI) from Invitrogen. Generation of HA-EPAC1 and CMV-myc-EPAC1-FLAG has been described [37]. pCAsalFLAG-GFP (GFP) was a generous gift from Professor Gwyn Gould, University of Glasgow, UK. pLV-CMV-ezrin-GFP and pLV-CMV-ezrinT567D-GFP were kind gifts from Professor Johannes Bos, University of Utrecht, Netherlands. p-eGFP-ezrin-T567A was a generous gift from Sabrina Marion, Institut Cochin, Paris, France.

### 2.2 Antibodies

The primary antibodies used were anti-EPAC1 (Clone 5D3, #4155; New England Biolabs; Ipswich, UK) and anti-ezrin (Santa Cruz Technologies, California, USA). Anti-Myc (#9E10), M2 monoclonal Flag (#F3165), anti-HA (#H9658) and anti-GFP were purchased from Sigma-Aldrich. InfraRed secondary antibodies (donkey anti-rabbit/anti-mouse 680 nm (#926-32213) and 700 nm (#926-32212)) were from Licor Biosciences (Nebraska, USA) and were imaged using the ODYSSEY® Sa Infrared Imaging System. Fluorescent secondary antibodies used for confocal imaging were Alexa-Fluor goat anti-rabbit/anti-mouse 488 nm and 568 nm (Alexa-Fluor, Invitrogen). Rhodamine Phalloidin was purchased from Invitrogen (Paisley, UK).

### 2.3 Cell culture

Human Embryonic Kidney (HEK293T) cell lines stably expressing either 3xFLAG-myc-CMV-26 vector (Sigma-Aldrich, UK) containing full-length human EPAC1, or vector alone, were prepared by Dundee Cell Products (Dundee, UK). COS1 and HEK293T cells were grown in Dulbecco's modified Eagle's medium (DMEM) 10% (v/v) foetal bovine serum (Sigma-Aldrich, UK), 2% (v/v) glutamine (Sigma-Aldrich, UK) and 2% (v/v) penicillin/streptomycin (Sigma-Aldrich, UK) and incubated at 37 °C in 5% (v/v) CO_2_. Selection of stable cell lines was maintained through addition of 400 μg/ml G418 (Sigma-Aldrich, UK) to the cell culture medium. Human umbilical cord endothelial cells (HUVECs) were grown in Endothelial Growth Medium MV2, supplemented with the MV2 supplement pack (Promocell, Heidelberg, Germany) and 1% (v/v) penicillin/streptomycin. Experiments using HUVECs were performed between Passages 1 and 6.

### 2.4 Cell transfection

Cells were grown to 50% confluence on ethanol sterilised Ø13 mm glass coverslips (for confocal analysis) or in 10 cm^2^ plates (for western blotting experiments). DNA constructs were then transfected into cells using Lipofectamine 2000 (Life Technologies; #116668-027) as per the manufacturer's instructions.

### 2.5 Immunofluorescent confocal microscopy

Cells were seeded at a density of 2 × 10^5^ on ethanol sterilised Ø13 mm glass coverslips and allowed to adhere overnight. Cells were then transfected as described and then stimulated with the indicated treatments prior to fixation with fixing buffer (3% (w/v) paraformaldehyde, 1% (w/v) sucrose, 1 mM CaCl_2_, 1 mM MgCl_2_ in PBS (37 mM NaCl, 2.7 mM KCl, 8 mM Na_2_HPO_4_, 1.46 mM KH_2_PO_4_, pH 7.4)). Coverslips were then quenched for 10 min in 50 mM NH_4_Cl in PBS, permeabilised for 4 min with 0.1% (v/v) Triton X-100 in PBS and then blocked with 0.02% (v/v) goat serum in 0.02% (w/v) filtered fish skin gelatine in PBS. Primary and secondary antibodies (anti-mouse/anti-rabbit FITC/rhodamine conjugates or rhodamine–phalloidin for actin stained cells) were incubated for 1 h at room temperature. Nuclei were visualised with 4′, 6-diamidino-2-phenylindole (DAPI, 10 μg/ml) or REDDOT (Cambridge Bioscience; #40061) for 20 min at room temperature. Coverslips were washed 3 times in block buffer between incubations. Coverslips were then mounted onto glass slides using Shandon Immu-Mount (Thermo Fisher Scientific, UK) and visualised using a 63 × Zeiss oil immersion objective, on a Zeiss confocal microscope (Carl Zeiss, Germany). Alexa Fluor® dyes (488 nm) and GFP fusion proteins were excited with an argon laser whereas 568 nm Alexa Fluor® dyes and rhodamine phalloidin were excited with a helium neon laser. Zeiss Pascal software was used to collect images, which were saved in the LSM file format and analysed using ImageJ software (http://rsbweb.nih.gov/ij/). For morphology assessments ImageJ wand tool was used to select cells from images. These were then measured for area and perimeter.

### 2.6 Förster Resonance Energy Transfer (FRET) measurements

For FRET experiments HEK293T cells stably transfected to express the EPAC-CAMP FRET sensor [32] were transferred into a modified Tyrode solution (140 mM NaCl, 3 mM KCl, 2 mM MgCl_2_, 2 mM CaCl_2_, 15 mM glucose, 10 mM HEPES, pH 7.2). To allow for adaptation to these new conditions, the cells were first kept for 10 min in a cell culture medium/Tyrode mix (33/66%), before they were finally transferred into the Tyrode solution. All applied substances were premixed into 150 μl Tyrode solution before addition to the cells. FRET measurements were performed with a Nikon Eclipse FN-1, equipped with an Opto-Led fluorescent light source (Cairn-research), a Dual-View beam-splitter (Optical Insights) and a CoolSnap HQ^2^ camera (Photometrix). Images were acquired with a 40 ×/0.8 numerical aperture long distance water dipping objective (Nikon). Excitation wavelength was 536 ± 25 nm, excitation/emission dichroic was 455 nm long pass. Emission light was splitted by a 505 nm longpass dichroic and filtered at 480 ± 15 nm for CFP emission and 535 ± 20 nm for YFP emission. Acquisition and analysis was performed using Optofluor (Cairn Research), which is identical to Metafluor (Molecular Devices). To reduce cell stress by illumination, long term experiments were performed at binning 2, which reduces the image resolution but increases signal intensity. All curves/values are background-subtracted and corrected for drifting baselines.

### 2.7 Dynamic mass redistribution assay

Wild type HEK293T-vector and HEK293T–EPAC1 cells were plated at 10,000 cells/well in 30 μl growth media on a corning Epic 384 well cell assay microplate (PerkinElmer), and then incubated overnight at 37 °C. Cells were then equilibrated for 2 h at room temperature and then a baseline measurement was recorded using an Enspire plate reader. The cells were then treated with varying concentrations of the EPAC1 antagonist, ESI-09 (Sigma Aldrich) or the PKA inhibitors, H-89 (Tocris) or KT5720 (Tocris), in the presence or absence of forskolin (Tocris) and rolipram (Tocris) or 007-(8-pCPT-O′-Me-cAMP) (Biolog). Dynamic mass redistribution measurements (DMR) were then taken every minute for 60 min. All treatments were made up in DMSO (Fisher Scientific) and diluted in 1 × HBSS (Life Technologies)/20 mM HEPES (Sigma Aldrich) assay buffer. For data analysis DMR readings from HEK293T-vector cells were subtracted from HEK293T–EPAC1 cells.

### 2.8 Western blotting

Cells were washed twice with ice cold PBS, lysed in ice cold lysis buffer (50 mM HEPES pH 7.5, 150 mM NaCl, 1% (v/v) Triton X-100, 0.5% (w/v) sodium deoxycholate, 0.1% (w/v) SDS, 10 mM sodium fluoride, 5 mM EDTA, 10 mM sodium phosphate) with protease inhibitor cocktail (Roche) and then cell debris was removed by centrifugation at 13,000 ×*g* for 20 min. The bicinchoninic acid assay [38] was then used to assess protein concentration of cleared lysates. Equal protein amounts were loaded and then separated on 7% and 12% (w/v) SDS PAGE gels and then transferred to nitrocellulose membranes with equal protein loading verified by Ponceau S staining. Membranes were then incubated for 1 h in block buffer (1% (w/v) skimmed milk powder in TBST (50 mM Tris, 150 mM NaCl, 0.05% (v/v) Tween 20)). Membranes were then incubated with primary antibodies at 4 °C overnight followed by incubation with InfraRed (donkey 700 nm and donkey 800 nm) secondary conjugated antibodies for 1 h at room temperature. InfraRed secondary antibodies were visualised using the ODYSSEY® Sa Infrared Imaging System (Licor Biosciences, Nebraska, USA).

### 2.9 Statistical analyses

Statistical significance was determined using one-way analysis of variance (ANOVA) with Tukey post-test.

## 3 Results

### 3.1 EPAC1 and PKA cooperate to promote cell spreading

To confirm previous observations that activation of endogenous EPAC can control cell spreading [3,18,33,34], COS1 and HUVECs, both of which express EPAC1, were stimulated with a combination of the adenylate cyclase (AC) activator, forskolin, and the type 4 phosphodiesterase inhibitor, rolipram (F/R), to elevate intracellular levels of cAMP. Additionally the EPAC selective cAMP analogue 8-pCPT-2′-O-Me-cAMP (007) [35] was employed in order to assess the specific role of EPAC over PKA. Treatment of COS1 (1 h) or HUVECs (2 h) with either F/R or 007 led to significant increases in cell size (Supplementary Figs. 1 and 2). The ability of 007 to induce cell spreading indicates that endogenous EPAC activation is sufficient to promote cell spreading in both cell lines. However, in contrast to what was observed in COS1 cells, there was significantly more cell spreading observed in HUVECs stimulated with F/R than 007 (Supplementary Fig. 2B). Furthermore, the enhanced cell spreading promoted by F/R coincided with a significant redistribution of actin into cortical actin bundles at the cell periphery, an effect that was not observed in 007-stimulated HUVECs (Supplementary Fig. 2C). This suggests that EPAC1 activation alone is not sufficient to promote maximal levels of cell spreading or cortical actin bundling in HUVECs, and that there is an additional requirement for PKA. Therefore cooperativity must exist between EPAC and PKA signalling pathways in HUVECs that underlies the cytoskeletal reorganisation required for maximal cell spreading.

**Fig. 1:**
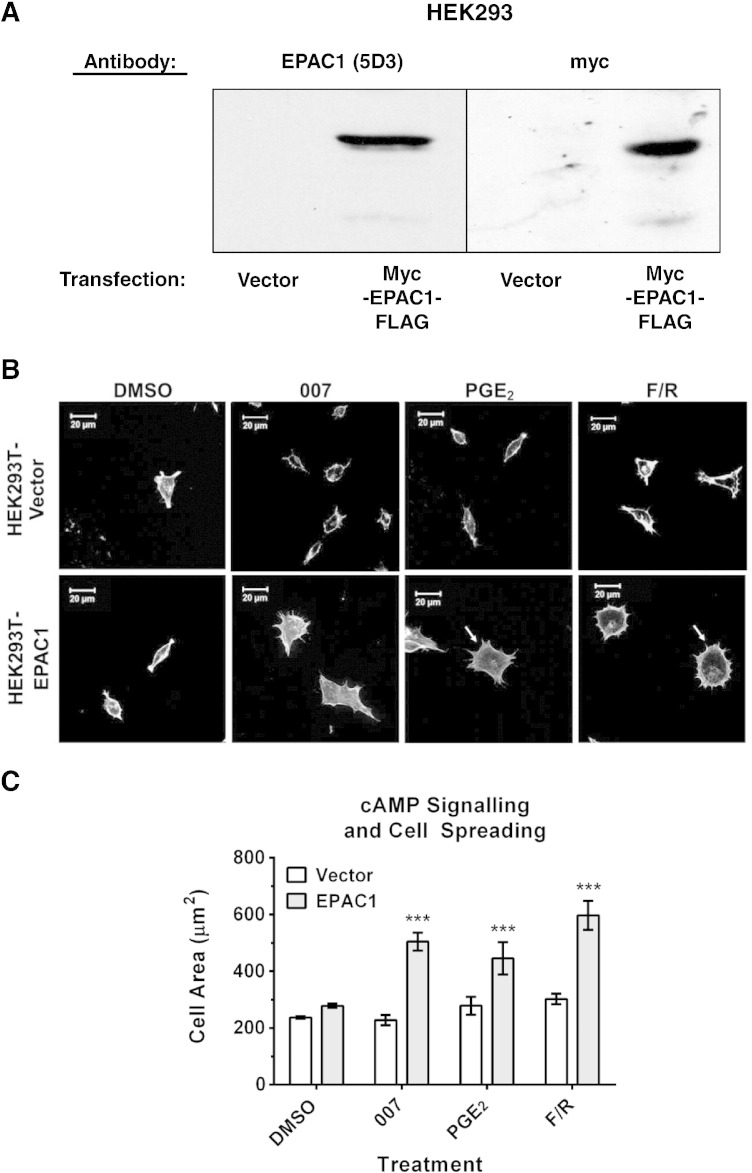
EPAC1 is required for cell spreading and cytoskeletal reorganisation in HEK293T cells. A) Cell extracts were prepared from HEK293T cells that had been stably transfected with either vector alone or myc- and FLAG-tagged EPAC1. Extracts were then immunoblotted with either anti-EPAC1 (*left*) or anti-myc antibodies (*right*). B) HEK293T–EPAC1 and HEK293T-vector cells were stimulated with either 007, prostaglandin E2 (PGE2) or F/R (10 μM, 60′) and then F-actin was visualised with rhodamine phalloidin. Arrows indicate actin polymerisation at the cell periphery. C) Cell areas were calculated from 10 random cell images (displayed as mean of 3 independent experiments ± SEM). *** = P < 0.001 (two way ANOVA).

To investigate this cooperativity further we generated a HEK293T cell line that stably expresses myc- and FLAG-tagged EPAC1 or vector alone. We found that HEK293T–EPAC1 cells, but not vector-containing cells, responded to the cAMP-elevating agents, prostaglandin E2 (PGE2) and F/R, and 007 with a significant increase in cell spreading (Fig. 1). Interestingly, as observed with HUVEC cells, cortical actin bundling occurred in response to PGE2 and F/R treatment, but not 007, in HEK293T–EPAC1, but not vector-only cells (Fig. 1). This suggests that there is a fundamental requirement for EPAC1 for cAMP-promoted cell spreading and cortical actin bundling in these cells. Moreover, although EPAC1 activation promotes cell spreading it is not sufficient to promote actin bundling, implicating an additional role for PKA in producing these effects.

**Fig. 2:**
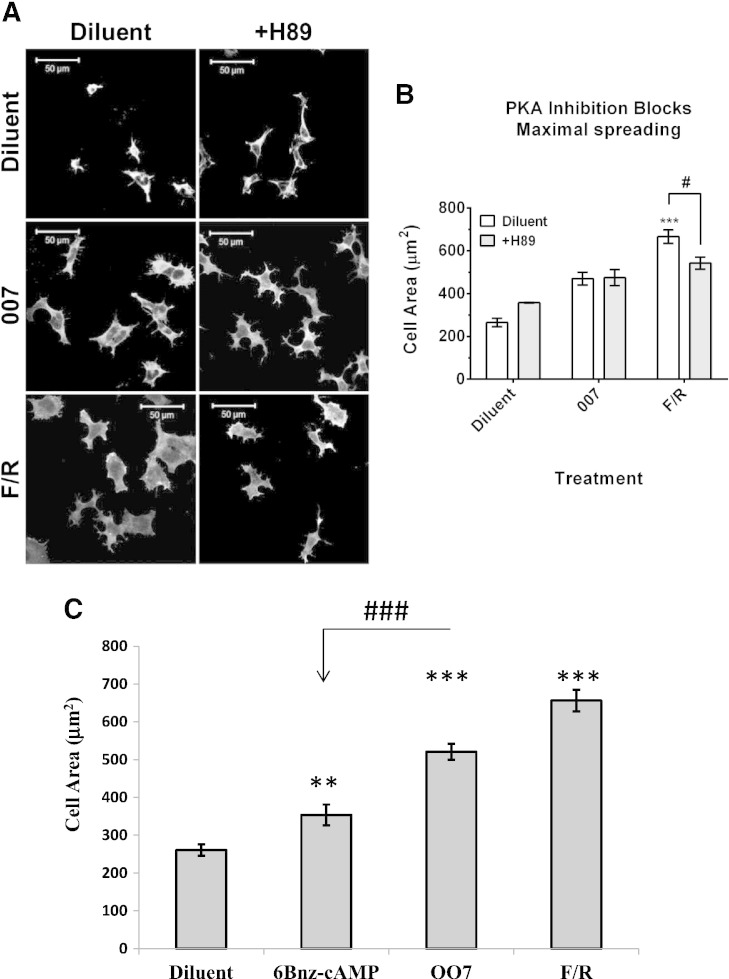
PKA inhibition inhibits cAMP but not EPAC1-mediated cell spreading. A) HEK293T–EPAC1 cells were pre-incubated with the PKA inhibitor H-89 (10 μM, 30′) and then stimulated with either F/R or 007 (10 μM, 60′). Cells were then probed using α-ezrin antibodies (white) to detect the cell body. B) Cell areas were calculated from 10 randomly acquired images and mean cell area from three independent experiments are shown (± SEM). Significance is indicated ***, p < 0.001, relative to diluent-treated cells, and #, p < 0.05, for H-89-treated cells, relative to F/R-treated cells. C) HEK293T–EPAC1 cells were stimulated with 6Bnz-cAMP (10 μM, 60′), 007 (10 μM, 60′) or F/R. Cell areas were then calculated from 10 images and mean cell area ± SEM (n = 3) is presented as a histogram. Significant increases in cell area relative to diluent-treated control cells are indicated, ***, p < 0.001, as are significant differences between 007- and 6-Bnz-cAMP-treated cells (###, p < 0.001).

EPAC1 and PKA have been observed to act synergistically to promote a range of cellular processes [36–40]. In order to test whether PKA cooperates with EPAC1 to promote cell spreading, HEK293T–EPAC1 cells were treated with 007 and F/R, in the presence or absence of the PKA inhibitor, H-89 [41]. We found that H-89 treatment had no effect on basal cell area or cell spreading induced by 007 (Fig. 2). However, cell spreading in response to F/R was significantly reduced to levels comparable with 007 stimulation, following H-89 treatment (Fig. 2B). This indicates that cooperativity exists between PKA- and EPAC1-regulated signalling pathways to promote maximal cell spreading following cAMP stimulation of HEK293T cells.

It should be noted, however, that when we measured EPAC1 activation in HEK293 cells (Supplementary Fig. 3) or SK-N-SH cells (results not shown), following stimulation with either 007 or F/R, we find that F/R always promotes a significantly greater degree of EPAC1 activation than 007 alone (Supplementary Fig. 3), suggesting that the larger increases in EPAC1 activation promoted by F/R my underlie the associated enhancement of cell spreading (Fig. 2). However, our data supports the idea that cell spreading is, in the first instance, EPAC1-dependent, as demonstrated in HEK293T–EPAC1 cells compared to HEK293-vector cells as seen in Fig. 1, and that the enhanced cell spreading seen with F/R treatment being attributable to PKA activation (Fig. 2).

It should be noted, however, that the PKA inhibitor, H-89, has been reported to have a number of off-target effects, including inhibition of PKC, ERK and AKT signalling pathways [42]; we therefore sought to determine the role of these kinases in controlling EPAC1-dependent cell spreading. A direct link between EPAC mediated signalling and AKT activation has been reported to control cytoskeletal reorganisation and adhesion in mesenchymal stem cells [43]. We therefore used the inhibitor of AKT activation, LY294002, to test the requirement for AKT in EPAC1-dependent cell spreading. Although 10 μM LY294002 effectively inhibited insulin-induced phosphorylation of AKT at a site indicative of activation (Ser 473) in EPAC1–HEK293 cells (Supplementary Fig. 4), it was unable to affect cAMP induced cell spreading in response to F/R in the same cells (Supplementary Fig. 4A and B). Similarly, EPAC1 activation has, controversially, been associated with activation of the ERK, MAPK cascade [15,43,44]. Indeed, we found that cAMP elevation with F/R is sufficient to induce ERK phosphorylation (Thr202/Tyr204) in HEK293T–EPAC1 cells (Supplementary Fig. 5C). Despite being activated in response to cAMP, inhibition of ERK activity with the MEK inhibitors, 1 μM AZD6244 and 1 μM PD184352, had little effect on EPAC1-dependent cell spreading (Supplementary Fig. 5A and B) indicating that although ERK is a downstream target for cAMP signalling this occurs independently to the changes in cell morphology associated with activation of EPAC1. This idea is supported by our observation that conventional (cPKC) and novel (nPKC) PKC isoforms are also not required for EPAC1-dependent cell spreading (Supplementary Fig. 6). We had previously reported that the activity of cPKCα and nPKCδ isoforms are required for EPAC1-dependent activation of gene expression in COS1 cells [45] and cyclic AMP-dependent ERK activation in HUVECs [46]. However it appears that these PKC isoforms do not play a role in EPAC1-promoted cell spreading in HEK293 cells. Accordingly activation of cPKCs and nPKCs, following treatment of EPAC1–HEK293 cells with 100 nM of the phorbol ester, PMA, lead to cell shrinkage, rather than spreading, and this was reversed by the PKC inhibitors Gö-6983 and Bisindolymaleimide I (Supplementary Fig. 6A and B). Hence, although PKC isoforms appears to play a role in controlling cell morphology in HEK293 cells, this appears to be mechanistically distinct from the changes induced following EPAC1 activation. In conclusion, it appears that cell spreading in response to cAMP elevation occurs independently of effects on AKT, ERK and PKC signalling and that it is unlikely that the actions of the inhibition H-89, on the suppression of cell F/R-induced cell spreading, occur independently of these pathways and is likely to be acting through inhibition of PKA.

Therefore, to provide further evidence that cell spreading is responsive to PKA activation in EPAC1 transfected cells, we stimulated cells with the PKA-selective agonist, N(*6*)-Benzoyl-cAMP (6Bz-cAMP), and measured cell spreading. We found that 6Bz-cAMP promoted a significant increase in cell spreading, which was significantly lower that the extent of cell spreading induced by 007 and F/R (Fig. 2C). This demonstrates that specific activation of PKA can promote cell spreading in EPAC1 expressing cells, although maximal induction of cells spreading is exerted by concomitant activation of PKA and EPAC1 in these cells, as occurs with F/R stimulation.

**Fig. 3:**
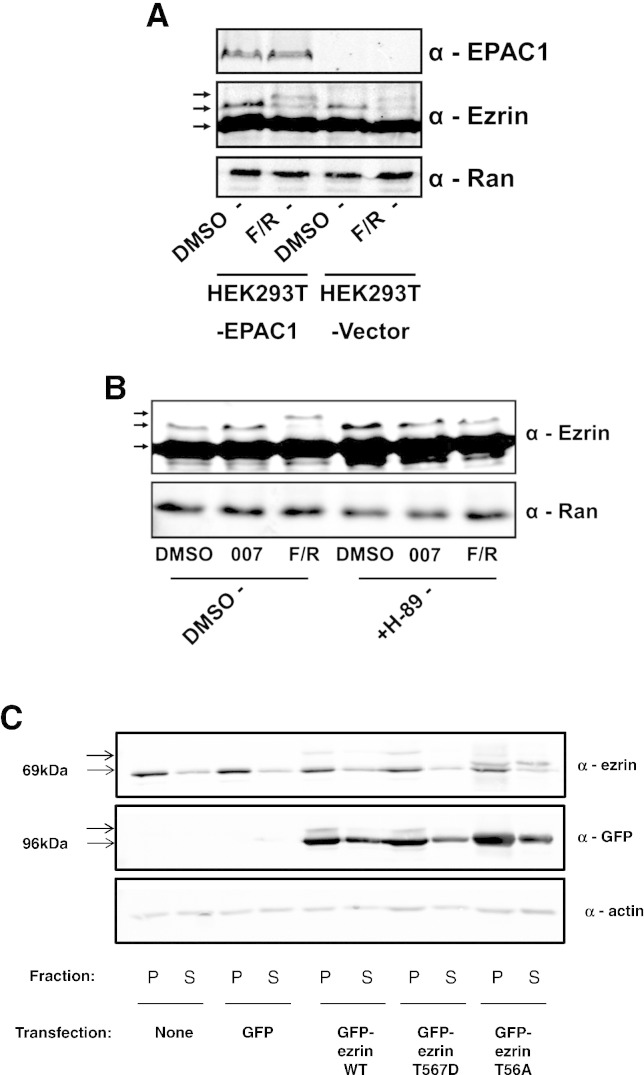
PKA activation promotes post-translational modification of ezrin. A) HEK293T–EPAC1 and HEK293T-vector cells were stimulated with F/R, as indicated, and then particulate fractions were prepared from cells and immunoblotted with anti-ezrin and anti-Epac1 antibodies. Arrows indicate the position of putative ezrin phospho-forms. Immunoblots are representative of an experiment done on 3 separate occasions. B) HEK293T–EPAC1 cells were pre-treated with H-89 (10 μM, 30′) and then stimulated with 007 and F/R, as indicated. Particulate cell fractions were then prepared and probed with anti-ezrin antibodies. Immunoblots are representative of an experiment done on 3 separate occasions. C) HEK-293T–EPAC1 was transfected with GFP-tagged wild type (WT), phospho-mimetic (T567D) and phospho-null mutants (T567A) of ezrin. Cells were then harvested and then separated into pellet and soluble fractions as described in the Materials and methods. Cell extracts were then immunoblotted with the indicated antibodies. Immunoblots are representative of an experiment done on 3 separate occasions.

We next examined the relationship between PKA and EPAC1 in the control of cell morphology by using an independent measure of cell spreading by measuring dynamic mass redistribution of cells (DMR) in response to pharmacological stimulation (Supplementary Fig. 7). This involved the use of Corning Epic technology to measure EPAC1-dependent cell-spreading, namely, the flattening and broadening of the cell as the cytoskeleton is redistributed closer to the plate. In all experiments the level of cell spreading detected in cell expressing vector alone served as a baseline to cell spreading in EPAC1-expressing cells. We found that stimulation of cells for up to 60 min induced a clear increase in DMR in both 007 and F/R-treated cells, however the level of DMR was consistently greater in F/R-treated than 007-treated cells (Supplementary Fig. 7A). Importantly, inhibition of PKA with H-89, or a chemically distinct PKA-inhibitor, KT5720, suppressed F/R-promoted DMR (Fig. 3A), but were not inhibitory in 007-stimulated cells and (Supplementary Fig. 7B and C). Moreover, the chemical inhibitor of EPAC activation, ESI-09, was also effective at inhibiting F/R-induced DMR (Supplementary Fig. 7A). Together these experiments indicate that maximal F/R-stimulated mass redistribution associated with cell spreading in HEK293T–EPAC1 cells is dependent on activation of both EPAC1 and PKA signalling routes by cAMP.

### 3.2 The actin-binding protein ezrin is mobilised in response to cAMP-elevation in HEK293T–EPAC1 cells

Results indicate that EPAC1-dependent mechanisms exist in HEK293T cells that allow the coordinated action of EPAC1- and PKA-activation to promote cell spreading and cortical actin bundling. One protein candidate for this integration of signalling is the actin cytoskeletal linker, ezrin, which has been shown to be essential for EPAC1-mediated cell spreading [3] and also for anchoring of PKA [47,48]. Ezrin also plays a central role in regulating the formation of the cell cortex and plasma membrane protrusions [47]. Considering this central role for ezrin in the control of plasma membrane dynamics and cytoskeletal organisation, we next examined whether ezrin is a target of cAMP-dependent signalling. HEK293T-vector and HEK293T–EPAC1 cells were treated with F/R and then particulate cell fractions were prepared and immunoblotted with anti-ezrin antibodies (Fig. 3A). Two distinct immunoreactive bands were detected on immunoblots comprising a low molecular weight band, representing ~ 80% of particulate ezrin, and a high molecular weight band, representing the remaining ~ 20% of ezrin protein. Following cAMP stimulation with F/R the higher molecular weight band underwent a band-shift, suggesting cAMP-dependent post-translational modification (Fig. 3A). The cAMP-induced band-shift was observed in both EPAC1- and vector-expressing cells (Fig. 3A), suggesting that post-translational modification of ezrin is not specifically linked to EPAC1 activity in HEK293T cells, and more likely to be a result of PKA activation. Indeed, it has been reported that PKA can regulate ezrin activity through phosphorylation of Ser66 [26] and Thr567 [31]. To test for a role of PKA in promoting the ezrin band-shift, cells were stimulated with 007 or F/R in the presence or absence of H-89. We found that EPAC1 activation with 007 did not induce a band-shift of Ezrin (Fig. 3B). In contrast, F/R provoked a robust band-shift that was ablated by co-treatment with H-89 (Fig. 3B), indicating that PKA activation is linked to the post-translational modification of ezrin, possibly through phosphorylation. It has previously been reported [31], that increases in the phosphorylation of ezrin on Thr567 in response to elevations in intracellular cAMP. To investigate whether phosphorylation of Thr567 is involved in the band-shift we observed following cAMP elevation we observed, we transfected HEK293T–EPAC1 cells with GFP-tagged wild type (WT), phospho-mimetic (T567D) and phospho-null mutants (T567A) of ezrin (Fig. 3C). We found that WT and phospho-mimetic GFP-tagged forms of ezrin appear to be constitutively phosphorylated, as evidenced by a high-molecular weight band-shifted form, whereas phospho-null ezrin exists as mainly a lower molecular-weight form (Fig. 3C). In addition, WT and the phospho-mimetic T567D mutant appear to induce a band-shift of endogenous ezrin in transfected cells, whereas the phospho-null mutant appears to suppress phosphorylation of endogenous ezrin (Fig. 3C). These results suggest that phosphorylation of Thr567 appears to contribute to the upward band-shift of ezrin following cAMP-stimulation of HEK293T–EPAC1 cells.

### 3.3 Ezrin is required for cAMP-mediated cell spreading in HEK293T Cells

**Fig. 4:**
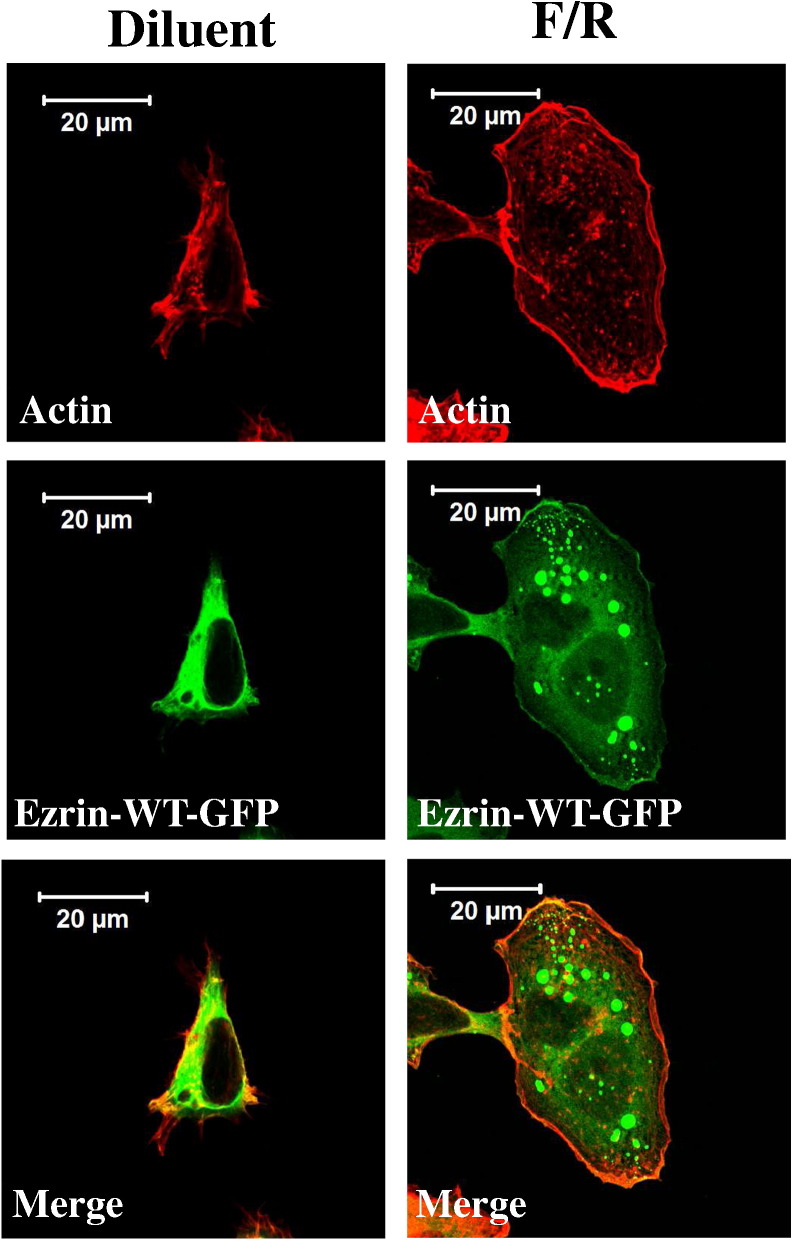
Ezrin accumulates at the plasma membrane in HEK293T cells. HEK293T–EPAC1 cells were transfected with a vector encoding GFP-tagged wild type ezrin (ezrin-WT-GFP) that were prepared for immunofluorescence as described in the Materials and methods. Cells were then stained with anti-actin antibodies and visualised by confocal microscopy. Results are representative of an experiment done on 3 separate occasions.

To further investigate a role for ezrin in coordinating PKA- and EPAC1-dependent signalling, we investigated the role of cAMP in determining the sub-cellular distribution of ezrin and other ERM proteins in HEK293T cells. Immunofluorescent staining with an antibody that detects the ERM family members ezrin, radixin and moesin demonstrated an accumulation of ERM immunoreactivity at the cell membrane following F/R-treatment of HEK293T–EPAC1 cells, but not HEK293T-vector cells (Supplementary Fig. 8), indicating that at least one ERM family member is responsive to cAMP in an EPAC1-dependent manner in these cells. Isoform-specific antibodies were next employed to determine which ERM family members were responsive to EPAC1 activation. Results showed that whereas radixin and moesin maintained a largely diffuse distribution, regardless of cell line or treatment applied (Supplementary Fig. 8). In contrast, following cAMP stimulation GFP-tagged ezrin appeared to become more diffuse within the cytosol and at the membrane, likely due to increased cell area and perimeter associated with isotropic cell spreading (Fig. 4). The accumulation of GFP-ezrin protein at the cell periphery, alongside actin, suggests that ezrin may be linked to cortical actin reorganisation following EPAC1 activation. Furthermore, the distribution of total ERM (Supplementary Fig. 8) and ezrin (Fig. 4) at the plasma membrane suggests that ezrin is the cAMP-responsive ERM protein in HEK293T–EPAC1 cells.

**Fig. 5:**
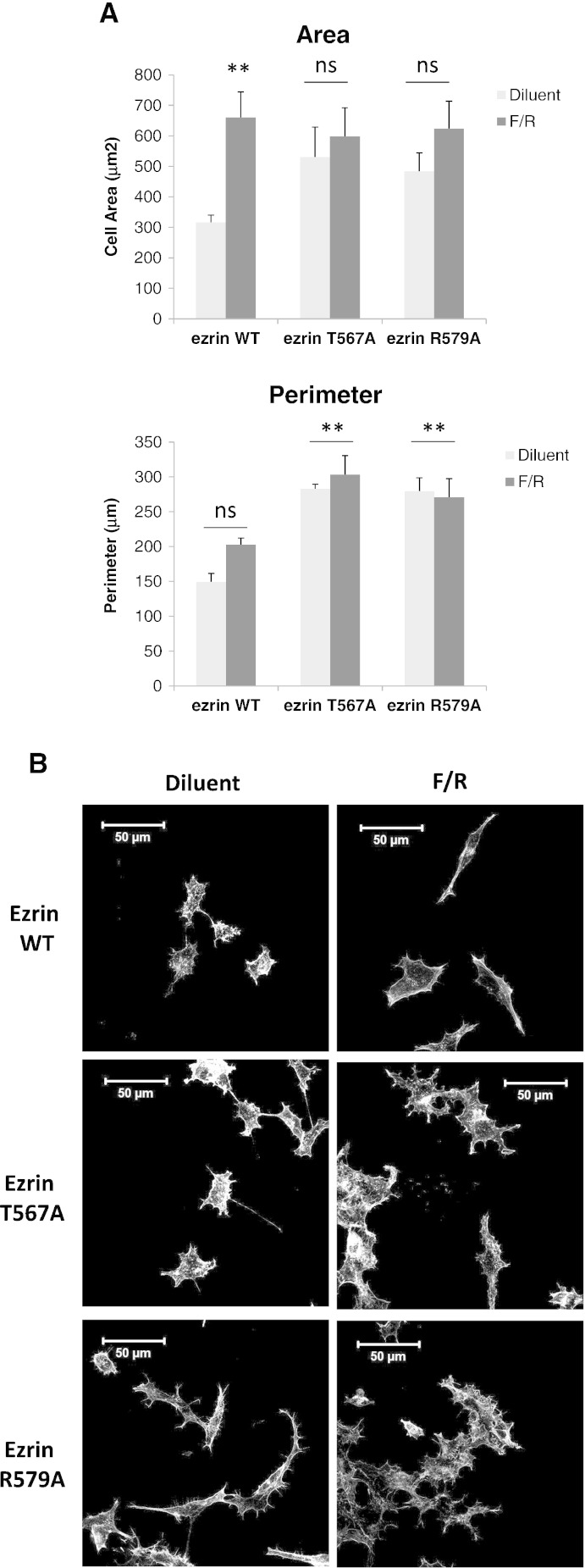
Dominant negative forms of ezrin induce cell spreading and arborisation in HEK293 cells independently of cAMP. A) HEK293T cells were transfected with GFP-tagged wild-type (WT) ezrin or dominant-negative ezrin (T567A) and ezrin (R579A) constructs. Cells were then stimulated with F/R for 60 min. GFP-expressing cells were then imaged using confocal microscopy and cell areas and perimeters were calculated (5 images per experiment, N = 3, ± SEM). Significant changes relative to diluent-treated cells are indicated, **, p < 0.01. B) Representative images of treated cells stained with rhodamine phalloidin.

Results indicate that ezrin is phosphorylated by PKA (Fig. 3) and mobilised to the cell periphery following EPAC1 activation (Fig. 4) in HEK293T cells. This suggests that ezrin may coordinate PKA- and EPAC1-dependent signals to control cell spreading. In order to investigate the role of ezrin in the control of cAMP-mediated cell spreading, HEK293T–EPAC1 cells were transfected with GFP-tagged ezrin-WT or two inactive mutants of ezrin; ezrin-T567A, which lacks the PKA phosphorylation site that is normally associated with ezrin activation [3], and ezrin-R579A, which has a reduced ability to couple the plasma membrane to the actin cytoskeleton [48]. Transfection of cells with ezrin-WT had no discernible effect on cell morphology or the cell-spreading response of HEK293T–EPAC1 cells following 007 (results not shown) or F/R treatment (Fig. 5). In contrast, the introduction of ezrin-T567A and ezrin-R579A mutants into cells produced dramatic changes in cell morphology, characterised by significantly higher cell perimeters (Fig. 5A), consistent with the formation of membrane projections (Fig. 5B). Furthermore, the inactive ezrin mutants suppressed the ability of F/R (Fig. 5A) and 007 (results not shown) to enhance cell spreading (Fig. 5A). These results indicate that phosphorylation of ezrin at Thr567 and interaction with actin are important for maintaining the basal cell morphology of HEK293T cells and that active ezrin is required for the subsequent actions of EPAC1 and PKA in the promotion of cAMP-induced cell spreading.

**Fig. 6:**
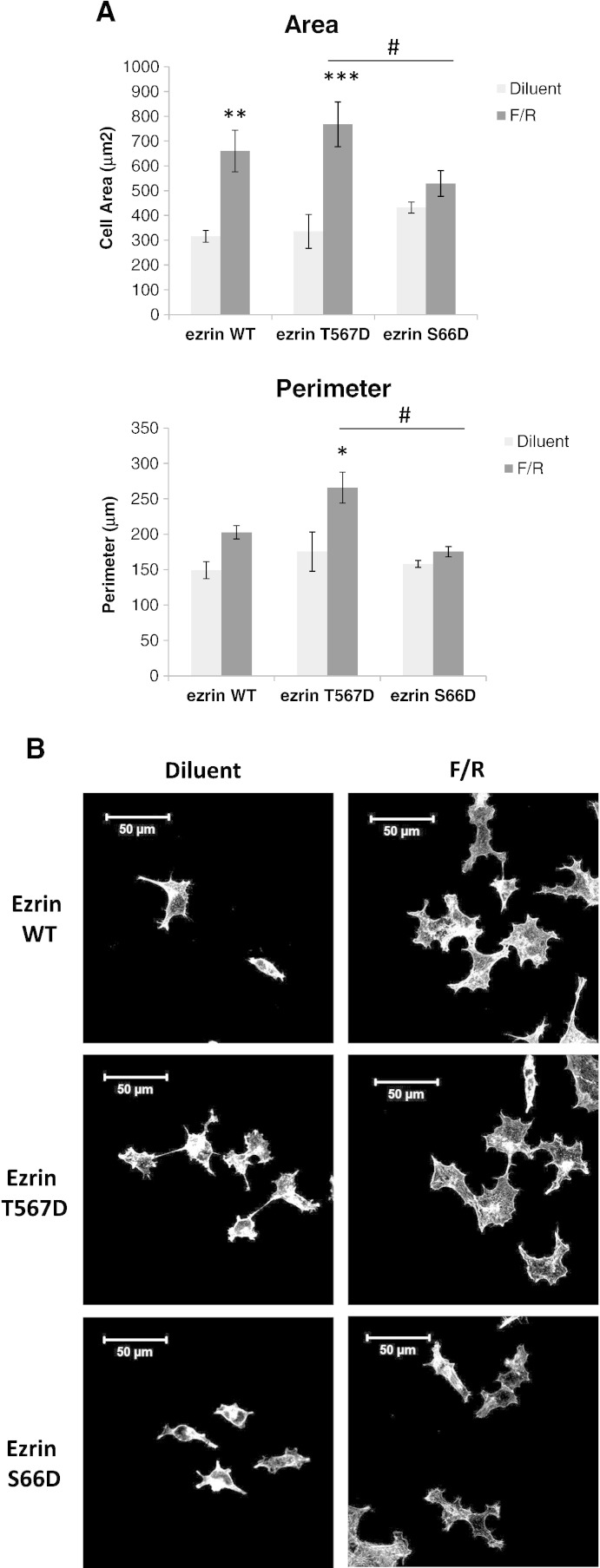
Effects of phospho-mimetic forms of ezrin on cAMP-promoted cell spreading. A) HEK293T cells were transfected with GFP-tagged ezrin-WT or phospho-mimetic forms of ezrin, ezrin-T567D and ezrin-S66D. Cells were then stimulated for 60 min with F/R. GFP-expressing cells were then imaged using confocal microscopy and cell areas and perimeters were calculated (5 images per experiment, N = 3, ± SEM). Significant changes relative to diluent-treated cells are indicated, **, p < 0.01 and ***, p < 0.001, as are significant changes relative to F/R-treated, ezrin-T567D cells. B) Representative images of treated cells stained with rhodamine phalloidin.

To further investigate the role of ezrin phosphorylation in the control of cAMP-mediated cell spreading, HEK293T–EPAC1 cells were transfected with GFP-tagged ezrin-T567D, a PKA phospho-mimetic mutant of ezrin [25]. We found that ezrin-T567D had no appreciable effect on cell spreading in response to EPAC1 and PKA activation with F/R (Fig. 6). This suggests that although phosphorylation of Thr567 is required for cAMP-induced cell spreading, as demonstrated by use of the T567A mutant (Fig. 5), it is not sufficient. We also investigated whether another PKA phosphorylation site in ezrin may also be required to promote cell spreading. In this respect, an additional PKA phosphorylation site has been identified in ezrin at Ser66 [26]. We found that cells expressing ezrin-S66D exhibited reduced cell spreading potential (Fig. 6), and is therefore probably not involved in the promotion of cell spreading by EPAC1 and PKA reported here. Together these results suggest that the ability of EPAC1 to promote PKA-dependent cell spreading requires interactions between ezrin and the actin cytoskeleton and basal phosphorylation of ezrin at Thr567, but not Ser66.

## 4 Discussion

Here we show that EPAC1 is absolutely required for cell spreading of HEK293T cells in response to cAMP stimulation. In addition, co-stimulation of EPAC1 and PKA with F/R induced further, PKA-dependent cell spreading and cortical actin bundling, over and above that induced by EPAC1 alone, indicating a secondary, synergistic effect of PKA on cell spreading in these cells. We conclude that EPAC1 may therefore gate the effects of PKA in HEK293T cells, perhaps by redistributing PKA into membrane ruffles, an effect previously observed to facilitate PKA association with Rho proteins at the cell surface [49].

In addition to cell spreading, the cooperativity between EPAC1 and PKA appears to underlie the reorganisation of the actin cytoskeleton following cAMP elevation; thus cortical actin bundling is observed only when both EPAC1 and PKA pathways are activated in concert. Recently, the ERM protein ezrin has been implicated as a regulator of EPAC1-mediated cell spreading [3]. Ezrin is able to regulate cortical actin structures and membrane dynamics [50] and both PKA and the Rho-activated kinase, ROCK, are involved in regulating ezrin activity by direct phosphorylation at key activation sites [26,31,51]. We report here that cAMP promotes PKA-dependent phosphorylation of ezrin in HEK293T cells. Although only a small fraction of total cellular ezrin was observed to undergo a band-shift, this is in agreement with previous reports suggesting that rapid turnover prevents the accumulation of ezrin phospho-forms [31]. We found that inhibition of ezrin phosphorylation at the PKA-target site, Thr567, or inhibition of ezrin interaction with the actin cytoskeleton had striking effects on cell morphology. This was associated with a large increase in the basal perimeter, consistent with the formation of multiple projections from the cell. Despite promoting an increase in cell area (Fig. 5A), this appeared to be due to contributions made by cell projections (Fig. 5B), rather than the uniform isotropic spreading observed in response to EPAC1 activation (Fig. 1). Indeed, the similarity in cell responses to the phospho-null ezrin mutant, ezrin-T567A, and the actin binding mutant, ezrin-R579A, in terms of cell morphology, suggest that cells expressing ezrin-T567A may no longer be able to form stable membrane–actin linkages. Indeed earlier reports have suggested that active ezrin plays a role in stabilising the plasma membrane and regulating the formation of cell projections [48]. The requirement for PKA-phosphorylation on ezrin-Thr567 for effective cAMP-promoted cell spreading (Fig. 3) suggests that EPAC1 requires PKA-promoted stabilisation of ezrin-regulated cell projections in order to mediate morphological change. An additional point to note is that ezrin has been reported to serve as an anchoring protein that recruits PKA to lipid rafts [52]. The recruitment of PKA by ezrin also appears to require Thr567, since the T567A mutant, but not the T567D mutant, is impaired in its ability to interact with the R1 regulatory subunit of PKA [52]. In the context of the current study it is difficult to ascertain the importance of this observation for the control of cell spreading, however it is clear that the increase in basal cell area promoted by inhibition of Thr567 phosphorylation reported here (Fig. 5) may also be linked to impaired intracellular targeting of PKA. Although it should be pointed out that the T567D mutant did not affect cell spreading in HEK293T–EPAC1 cells (Fig. 6), whereas this mutant should still retain the capacity to interact with PKA and, therefore, targeting of PKA by ezrin, at least to lipid rafts. Targeting of PKA by ezrin may therefore not impact greatly on the control of cell spreading by EPAC1, as reported here.

Overall, we propose that ezrin is involved in the cell spreading response of HEK293T–EPAC1 cells following cAMP stimulation by promoting isotropic growth through the stabilisation of the cell membrane and limiting actin rich projections. PKA appears to regulate this function by promoting Thr567 phosphorylation and the accumulation of ezrin at actin rich membrane ruffles subsequent to EPAC1 activation. In this regard ezrin has been shown to affect Rho GTPase signalling by anchoring it to cell projections (Ivetic and Ridley, 2004). In agreement with this we have found that inhibition of ROCK, inhibits cell spreading in response to F/R treatment of HEK293T–EPAC1 cells (Supplemental Fig. 9), suggesting a possible link between EPAC1, PKA-promoted ezrin distribution and ROCK activity in the promotion of maximal cell spreading by cAMP.

## Conflict of interest

The authors have no conflict of interest.

## Appendix A Supplementary data

Supplementary Fig. 1. EPAC activation promotes cell spreading in COS1 cells. A) COS1 cells were treated with either diluent (DMSO), 10 μM 8-pCPT-2′-O-Me-cAMP (007) or 10 μM forskolin plus 10 μM rolipram (F/R) for 60 min and then stained with rhodamine phalloidin to detect F-Actin. B) Mean cell areas were calculated from ten randomly acquired images (minimum 50 cells/image). Cell areas from 3 separate experiments are shown (mean ± SEM). Statistical significance is indicated; ***p < 0.001 (ANOVA). Supplementary Fig. 2. Effect of EPAC activation on cell spreading and actin bundling in HUVECs. A) HUVECs were treated with either diluent (DMSO), 10 μM 007 or F/R for 120 min and then stained with rhodamine phalloidin. B) Cell areas were calculated from ten randomly acquired images (minimum 50 cells/image) from 3 separate experiments (mean ± SEM). Statistical significance (ANOVA) is indicated; *p < 0.05 and ***p < 0.001, relative to DMSO-treated cells, and #, p < 0.05, for F/R-treated cells relative to 07-treated cells. C) Cortical actin bundling was calculated from line scans taken across the longest cell axis. The percentage of actin found in the outermost 10% of both sides of 10 cells was normalised to total cell fluorescence to give the percentage of cortical actin (mean ± SEM). Statistical significance is indicated; *p < 0.05 (ANOVA). NS, indicated no significance. Supplementary Fig. 3. Analysis of EPAC1 activation following stimulation of HEK293 cells with 007 and F/R. Live HEK293 cells that had been stably transfected with aCFP-EPAC-YFP reporter construct [32] were stimulated with 10 μM 8-pCPT-2′-O-Me-cAMP (007) or 10 μM forskolin plus 10 μM rolipram (F/R) for the indicated time points. The percentage change in FRET activity (indicating EPAC activation) is displayed in graphical form. Supplementary Fig. 4. EPAC1-promoted cell spreading occurs independently of AKT activation. A) HEK293T–EPAC1 cells were stimulated for 60 min with either 10 μM Forskolin plus 10 μM Rolipram (F/R) or 1 μM insulin in the presence or absence of the AKT inhibitor, 10 μM LY294002, and then immunostained with anti-total ezrin antibodies and imaged by confocal microscopy. B) Changes in cell area from 5 randomly acquired images (minimum 30 cells) were determined as described in the Materials and methods and presented as a histogram in the *lower panel* (mean ± S.E.M.; n = 3). C) Following stimulation cell extracts were prepared and immunoblotted with antibodies that recognise total AKT or pAKT (Ser 473) as indicated. Supplementary Fig. 5. EPAC1-induced cell spreading occurs independently of ERK activation. A) Representative images of anti-ezrin stained HEK293T–EPAC1 cells following 60 min stimulation with 10 μM Forskolin plus 10 μM Rolipram (F/R) in the presence or absence of the MEK inhibitors AZD6244 (1 μM) or 1 μM PD184352 (1 μM). B) Changes in cell area from 5 randomly acquired images (minimum 30 cells) were determined as described in the Materials and Methods and presented as a histogram (mean ± S.E.M.; n = 3). C) Following stimulation cell extracts were prepared and immunoblotted with anti-pERK (Thr202/Tyr204), anti-total ERK and anti-pCREB (Ser 133) as indicated. Supplementary Fig. 6. EPAC1-dependent cell spreading occurs independently of activation of conventional and novel PKC activation. A) HEK293T–EPAC1 cells were stimulated for 60 min with either 10 μM Forskolin plus 10 μM Rolipram (F/R) or 100 nM of the conventional and novel PKC activator, PMA, in the presence or absence of the PKC inhibitors, 10 μM Gö6983 or 10 μM bisindolylmaleimide I (Bis I), as indicated. Cells were then visualised by confocal microscopy after immunostaining with anti-ezrin antibodies. B) Changes in cell area from 5 randomly acquired images (minimum 30 cells) were determined as described in the Materials and methods and presented as a histogram (mean ± S.E.M.) Statistically significant increases in cell area relative to diluent-treated cells are indicated ***, p < 0.001 (n = 3) as are significant decreases relative to F/R-treated cells, ^##^, p < 0.01 and ^###^, p < 0.001 (n = 3) respectively. Supplementary Fig. 7. Dynamic mass redistribution in HEK293T–EPAC1 cells. HEK293T-vector and HEK293T–EPAC1 were seeded on to Corning Epic microplates and then stimulated for up to 60 min with either F/R or 007 in the presence or absence of the EPAC inhibitor, 10 μM ESI-09 (A), or the PKA inhibitors, 10 μM H-89 or 10 μM KT5720 (B and C). Changes in dynamic mass reorganisation (DMR) were then measured using a charge-coupled detection microplate reader. Supplementary Fig. 8. ERM proteins accumulate at the plasma membrane in HEK293T–EPAC1 following cAMP stimulation. HEK293T–EPAC1 and HEK293T-vector cells were prepared for immunofluorescence (Materials and methods). Cells were stained with anti-EPAC1 antibodies to confirm expression (not shown) and for ERM proteins with the indicated antibodies. Supplementary Fig. 9. The ROCK inhibitor Y27632 attenuates cAMP-mediated cell spreading in HEK293T cells. A) HEK293T-vector and HEK293T–EPAC1 cells were stimulated for 60 min with F/R in the presence or absence of the ROCK inhibitor, 10 μM Y27632. Cells were then stained with rhodamine phalloidin and actin visualised by confocal microscopy. Arrows indicate the formation of elongated protrusions. B) Cell areas were calculated and presented as a histogram (mean ± SEM, n = 3). Significant differences relative to DMSO-treated cells are indicated, ***, p < 0.001, as are significant changes relative to F/R-treated, EPAC1-expressing cells, #, p < 0.05 (ANOVA).
