# Supplementary material for: Phosphorylation of ezrin on Thr567 is required for the synergistic activation of cell spreading by EPAC1 and protein kinase A in HEK293T cells
Source: Biochim Biophys Acta. 2015 Jul;1853(7):1749–58. doi: 10.1016/j.bbamcr.2015.04.009 (PMC4547084; doi:10.1016/j.bbamcr.2015.04.009)

Supplementary Figure 1

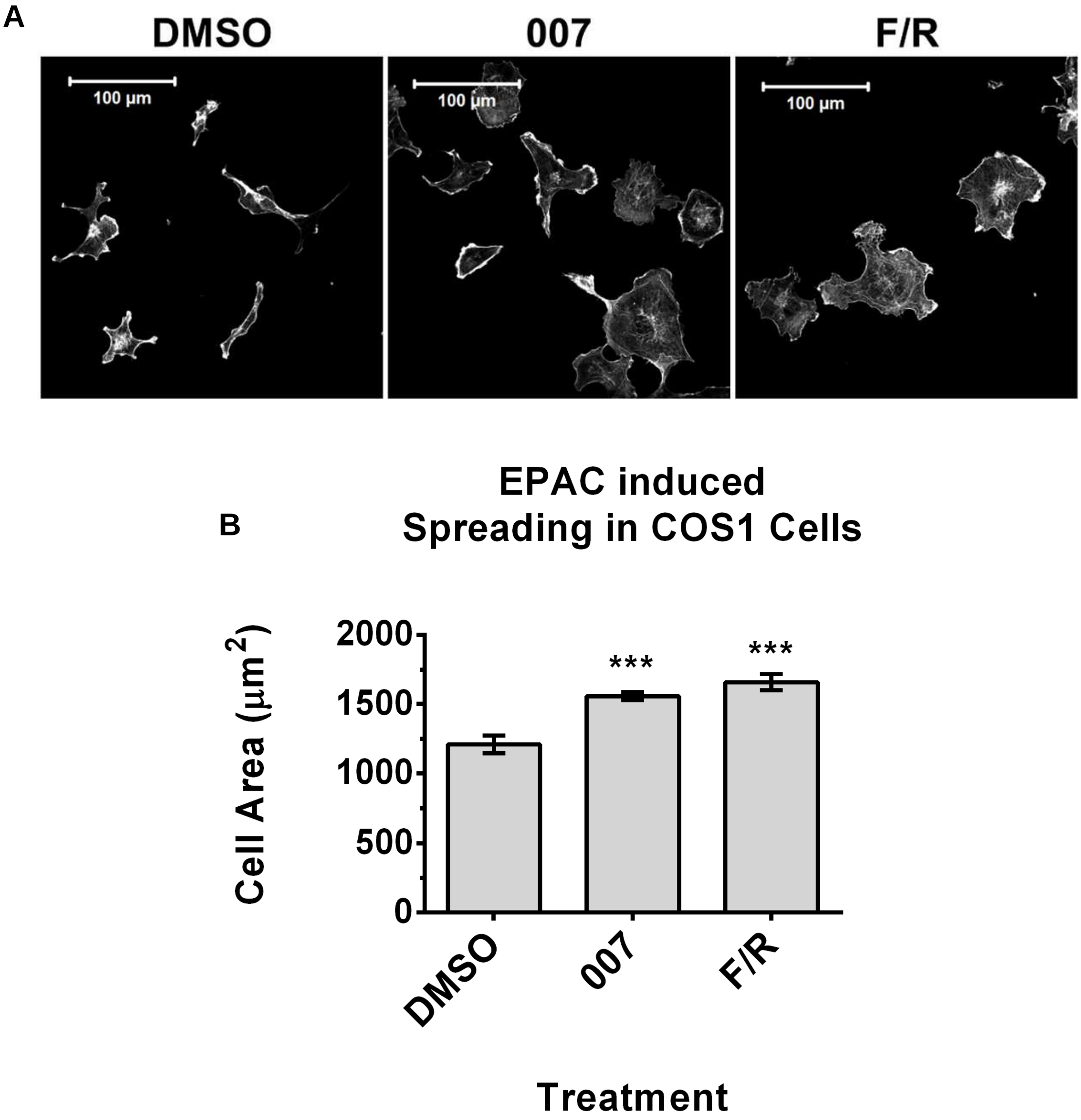

Supplementary Figure 2

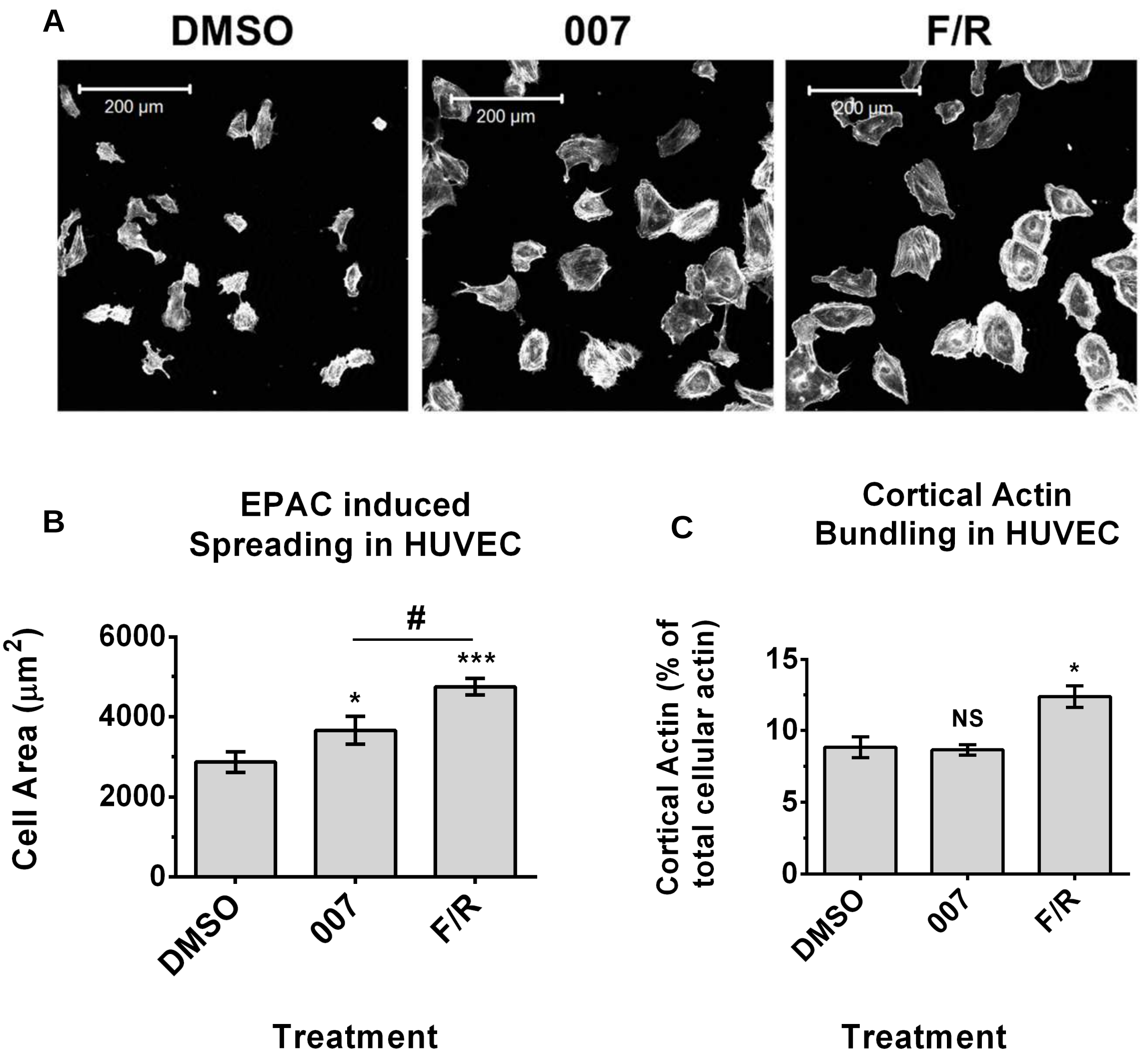

Supplementary Figure 3

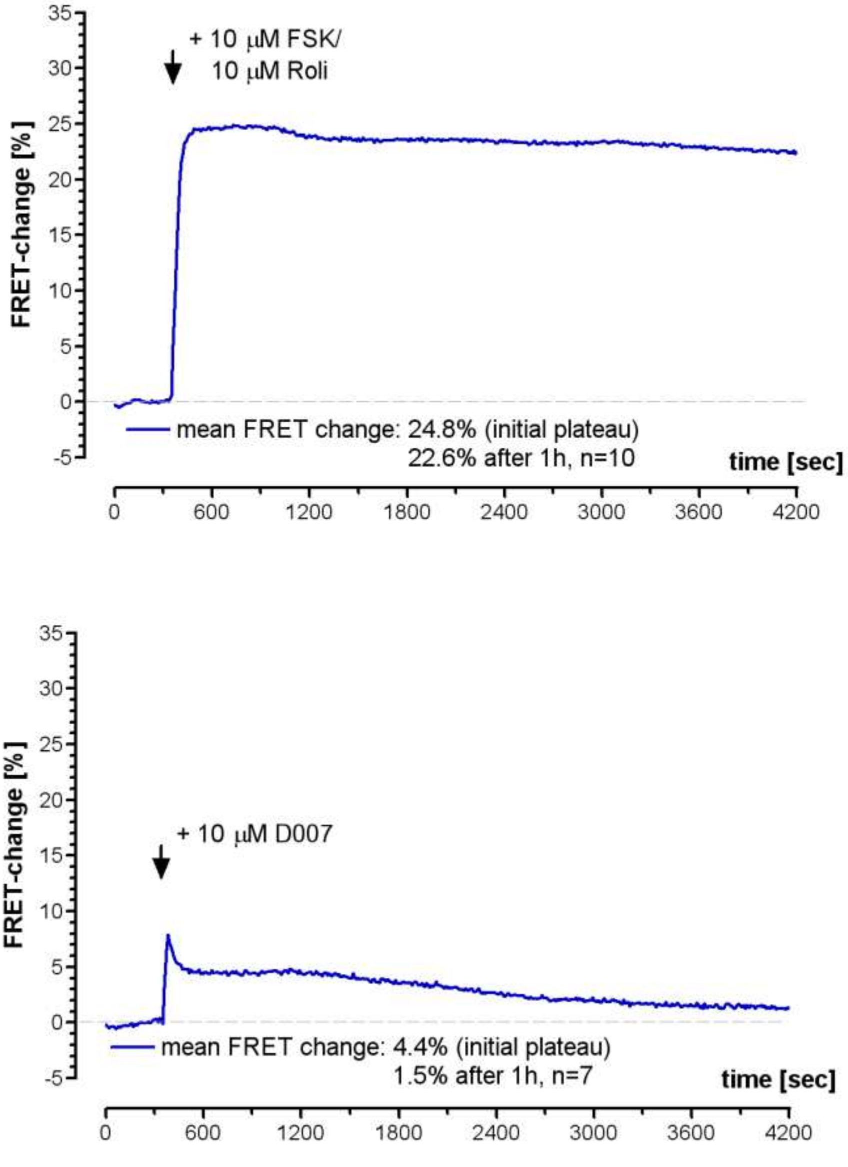

Supplementary Figure 4

A)

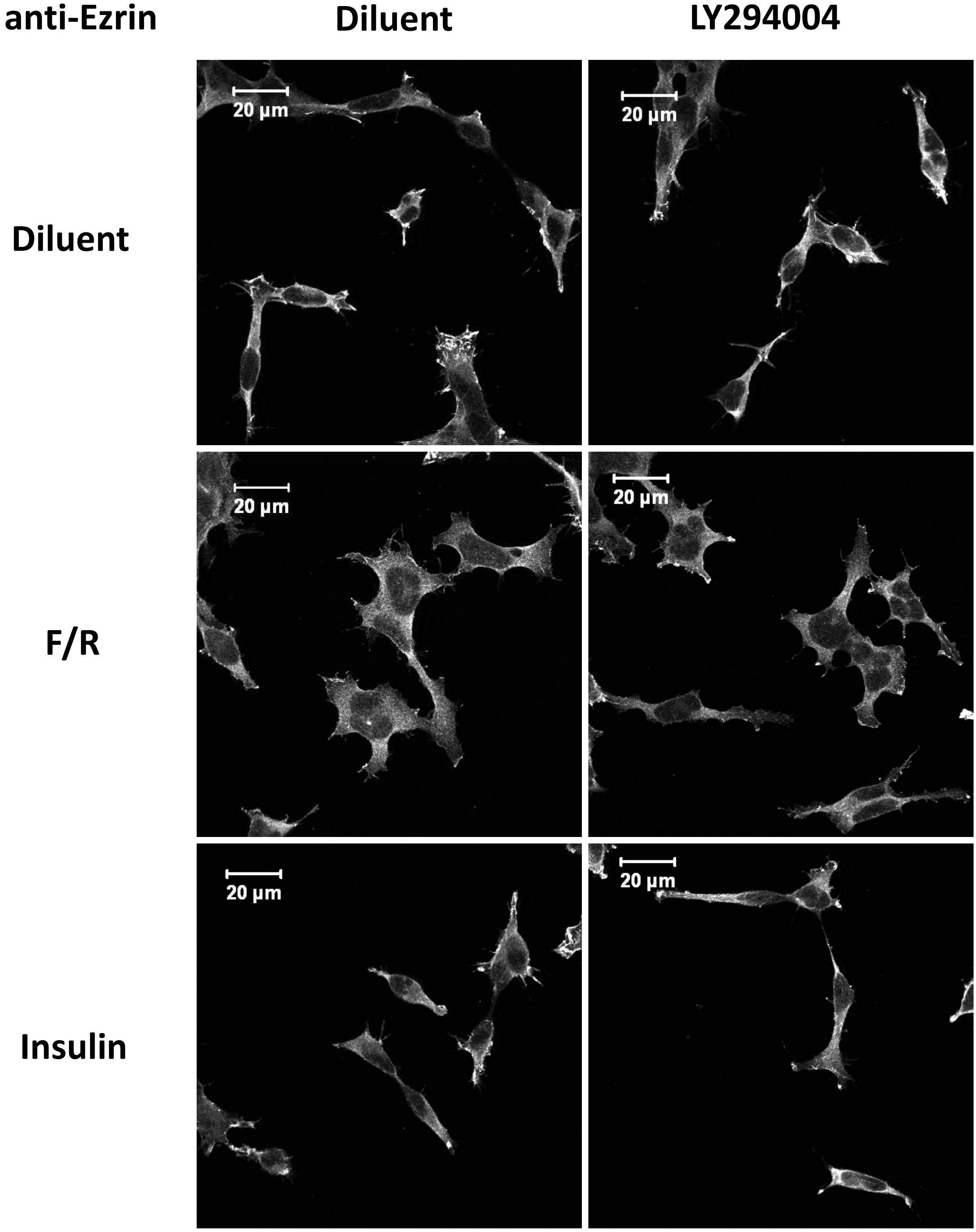

Supplementary Figure 4

B)

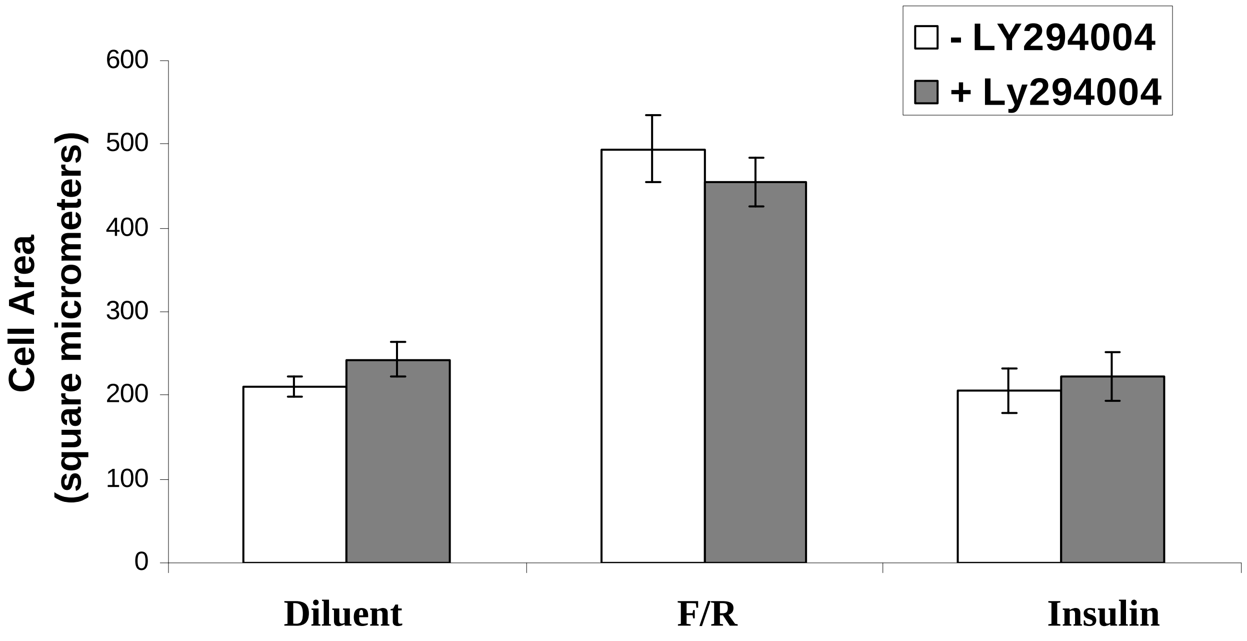

C)

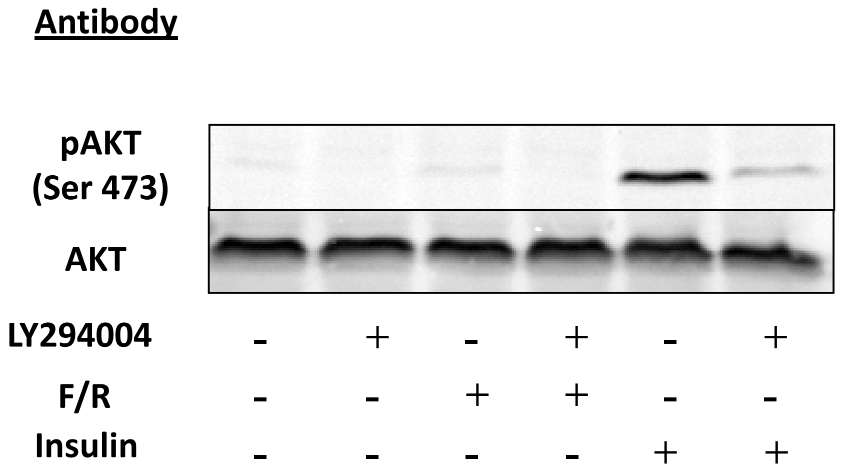

Supplementary Figure 5

A)

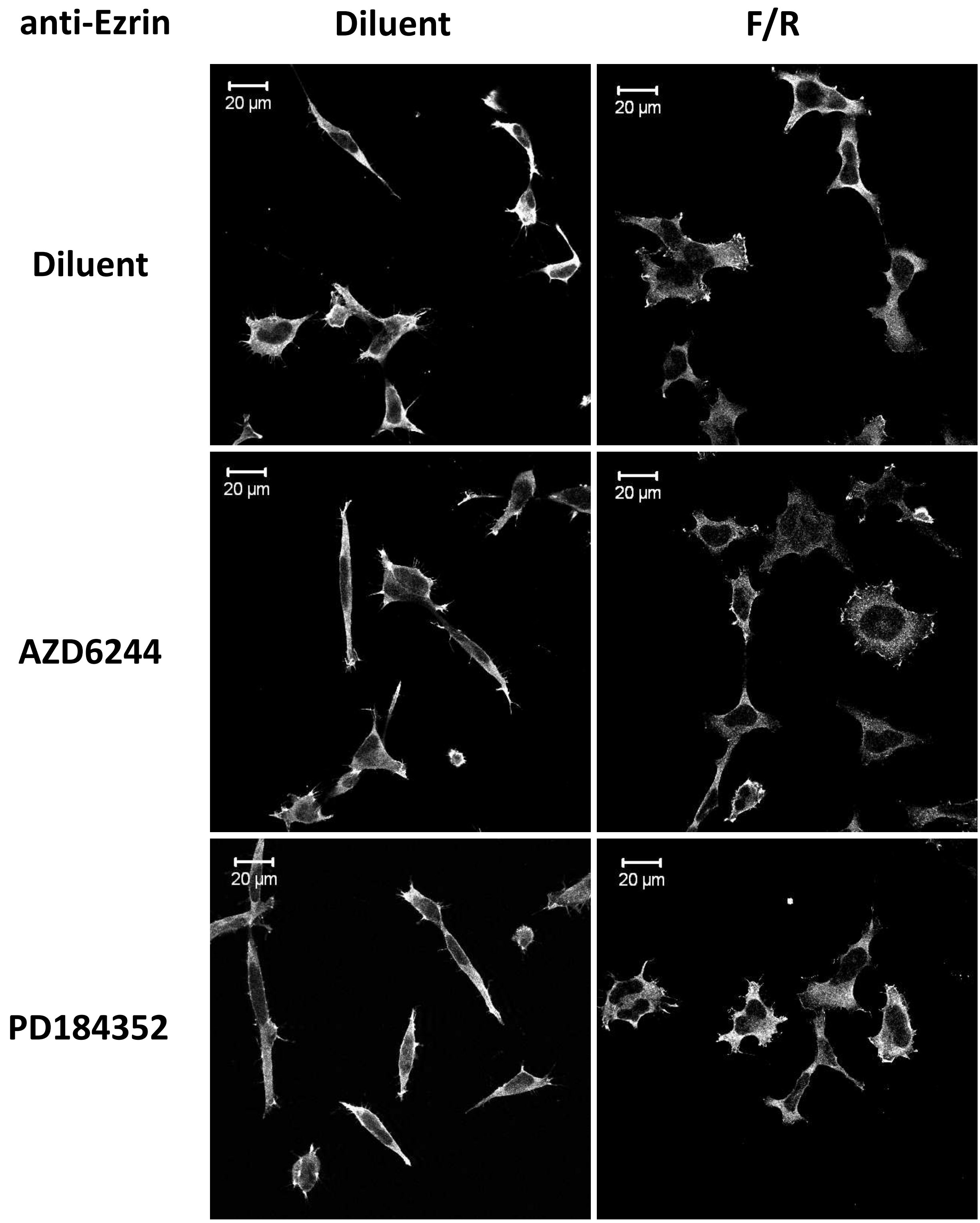

Supplementary Figure 5

B)

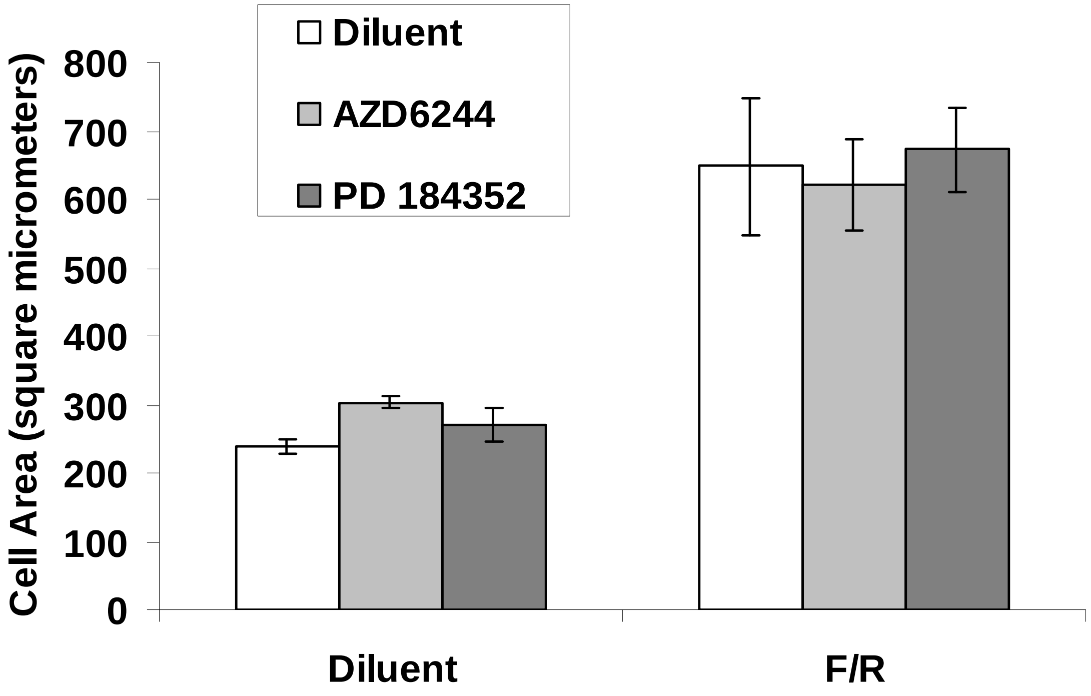

Supplementary Figure 5

C)

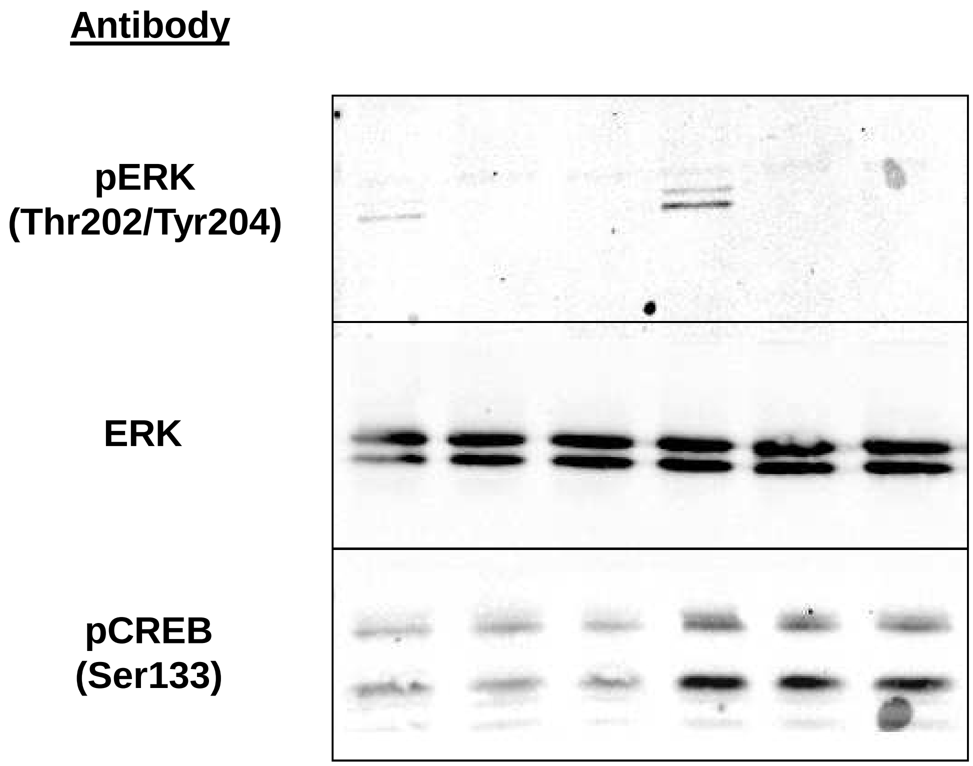

**F/R**	-	-	-	+	+	+
**AZD6244**	-	+	-	-	+	-
**PD 184352**	-	-	+	-	-	+

Supplementary Figure 6

A)

anti-Ezrin

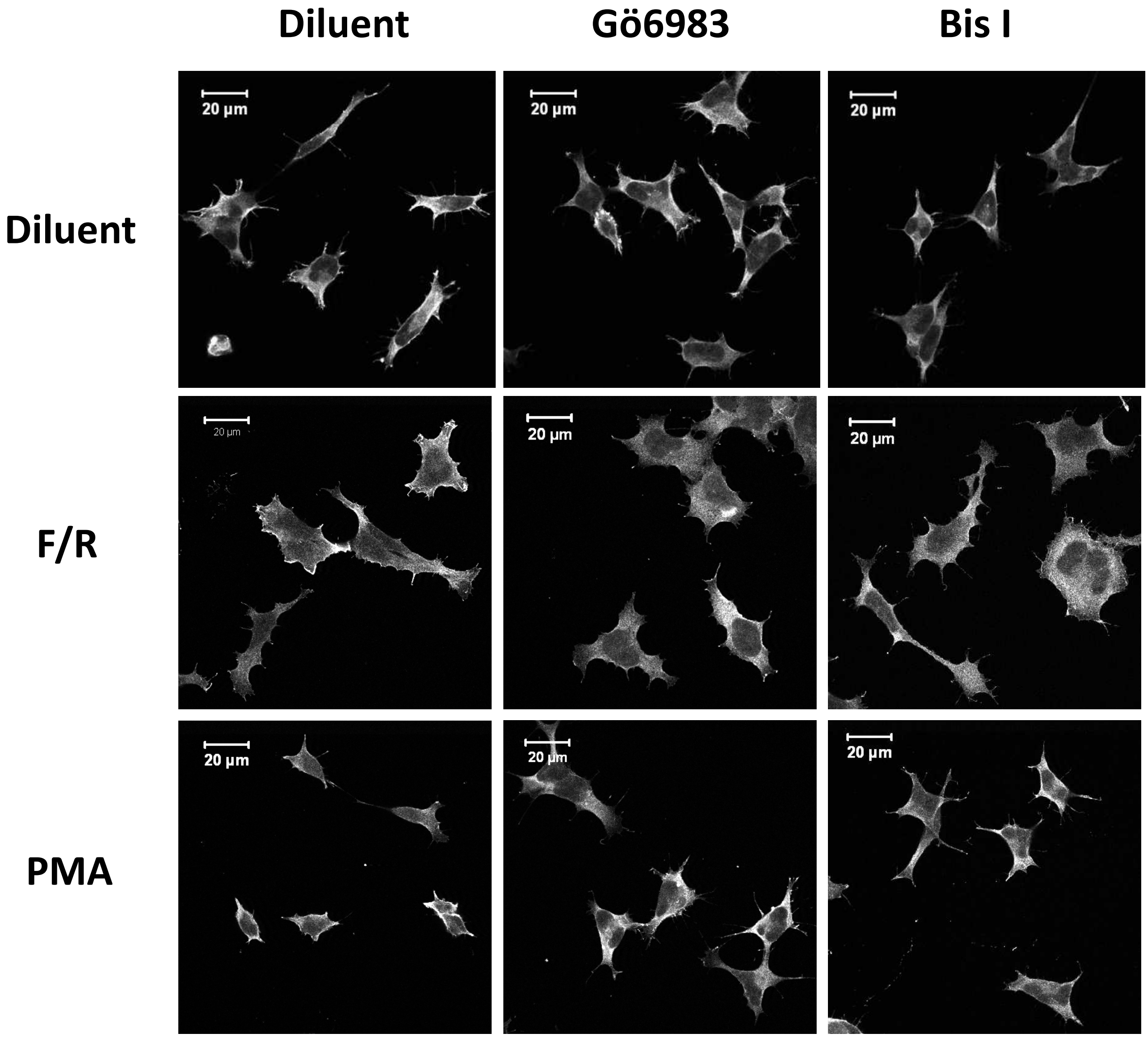

Supplementary Figure 6

B)

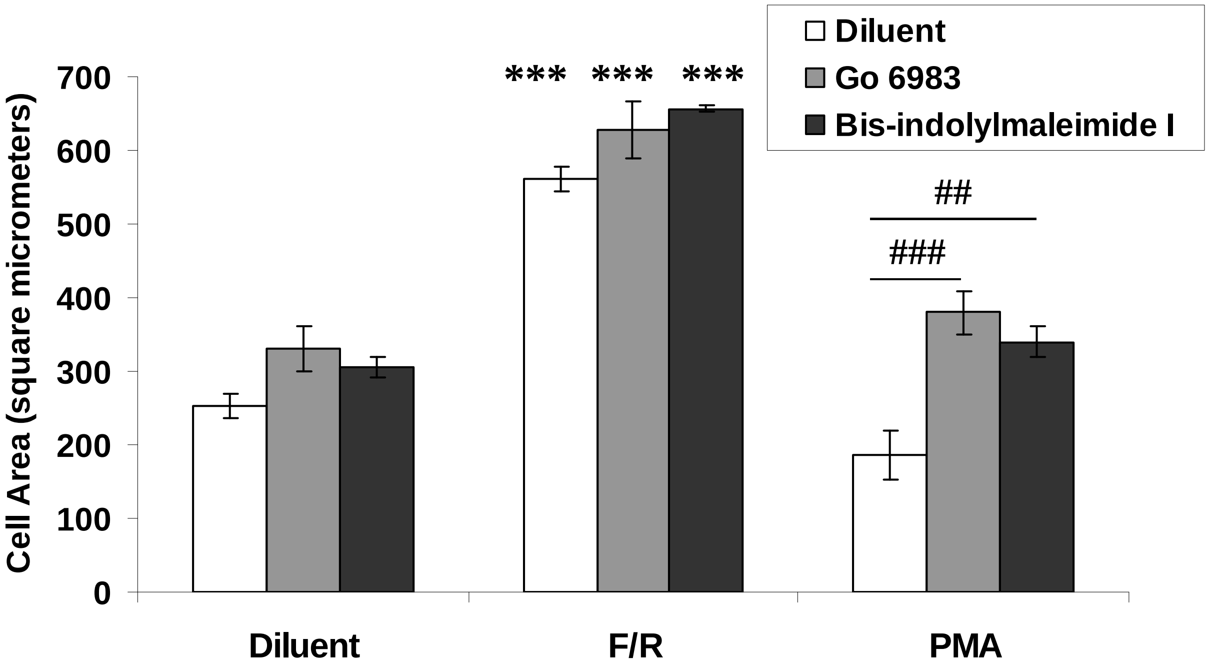

Supplementary Figure 7

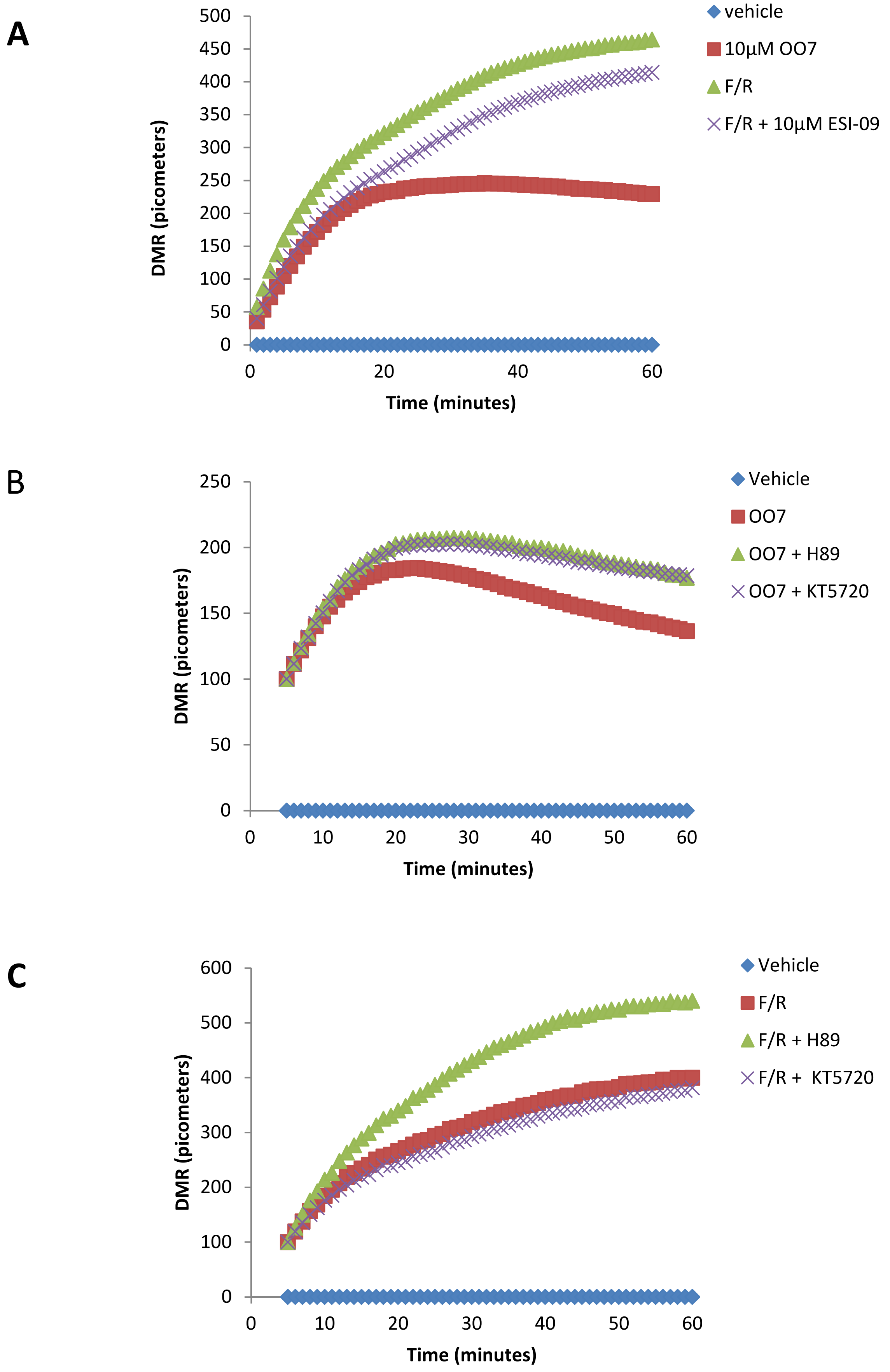

Supplementary Figure 8

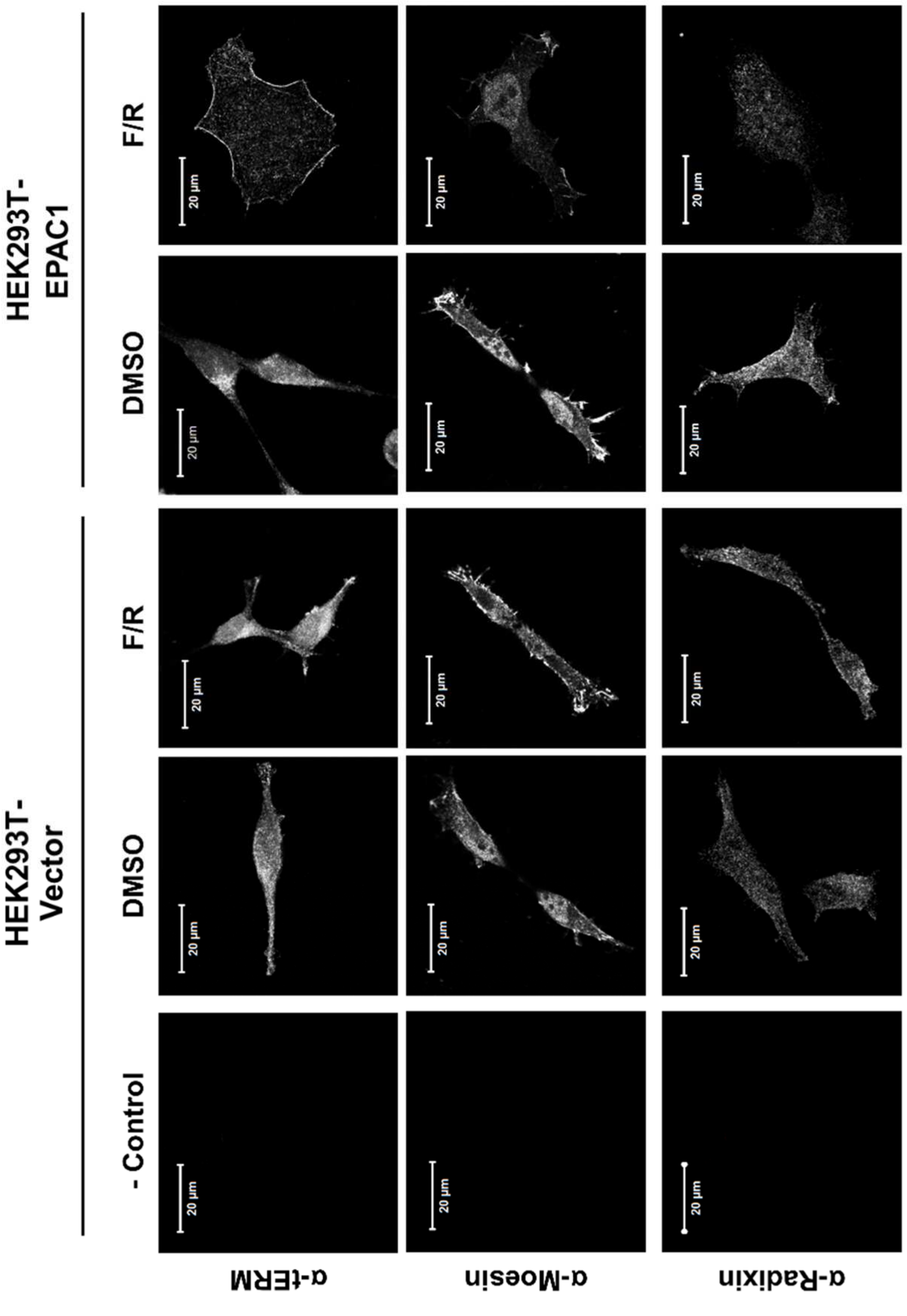

Supplementary Figure 9

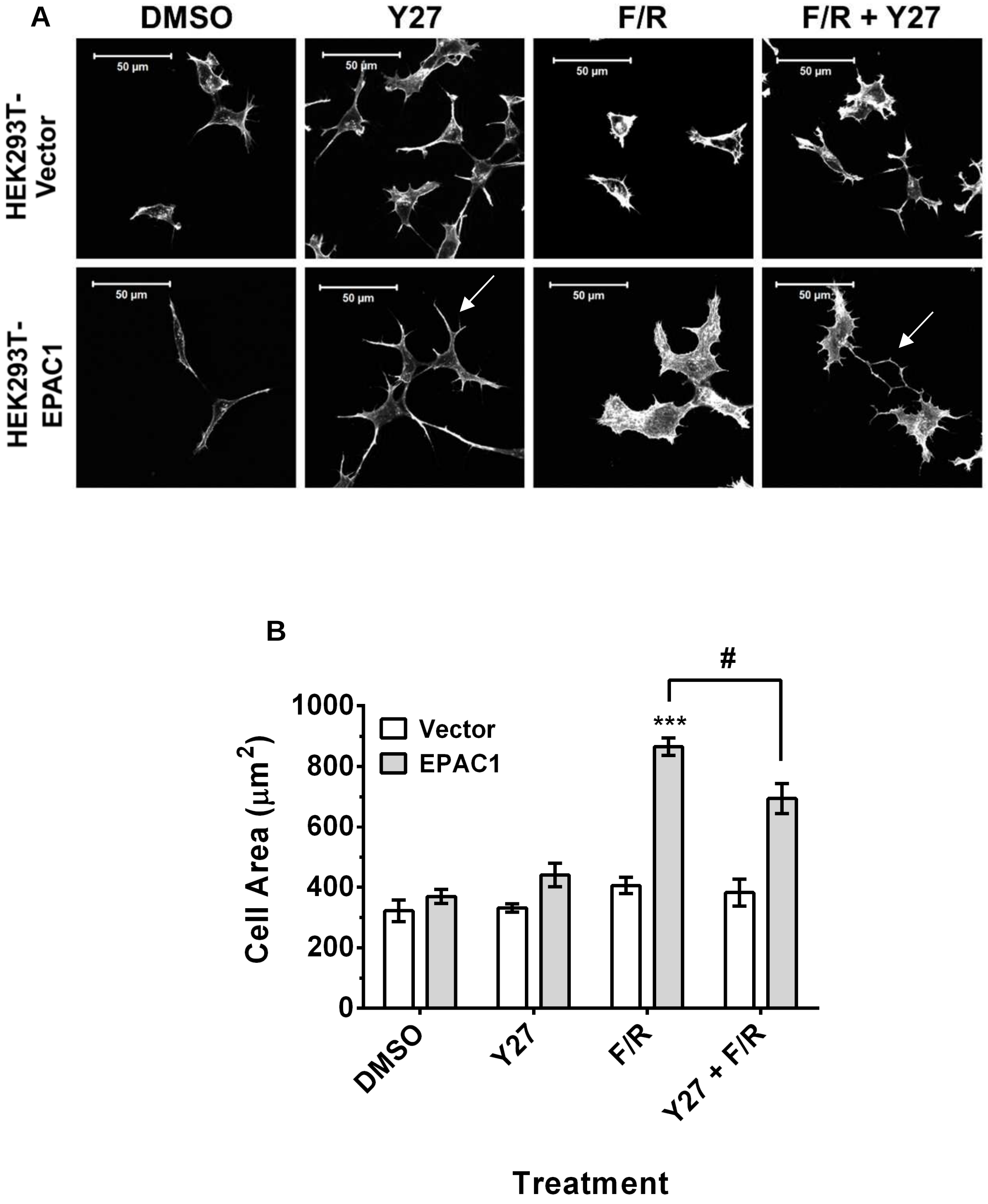

Supplement: Supplementary file 1 — Supplementary Fig. 1. EPAC activation promotes cell spreading in COS1 cells. A) COS1 cells were treated with either diluent (DMSO), 10 μM 8-pCPT-2′-O-Me-cAMP (007) or 10 μM forskolin plus 10 μM rolipram (F/R) for 60 min and then stained with rhodamine phalloidin to detect F-Actin. B) Mean cell areas were calculated from ten randomly acquired images (minimum 50 cells/image). Cell areas from 3 separate experiments are shown (mean ± SEM). Statistical significance is indicated; ***p < 0.001 (ANOVA). Supplementary Fig. 2. Effect of EPAC activation on cell spreading and actin bundling in HUVECs. A) HUVECs were treated with either diluent (DMSO), 10 μM 007 or F/R for 120 min and then stained with rhodamine phalloidin. B) Cell areas were calculated from ten randomly acquired images (minimum 50 cells/image) from 3 separate experiments (mean ± SEM). Statistical significance (ANOVA) is indicated; *p < 0.05 and ***p < 0.001, relative to DMSO-treated cells, and #, p < 0.05, for F/R-treated cells relative to 07-treated cells. C) Cortical actin bundling was calculated from line scans taken across the longest cell axis. The percentage of actin found in the outermost 10% of both sides of 10 cells was normalised to total cell fluorescence to give the percentage of cortical actin (mean ± SEM). Statistical significance is indicated; *p < 0.05 (ANOVA). NS, indicated no significance. Supplementary Fig. 3. Analysis of EPAC1 activation following stimulation of HEK293 cells with 007 and F/R. Live HEK293 cells that had been stably transfected with aCFP-EPAC-YFP reporter construct [32] were stimulated with 10 μM 8-pCPT-2′-O-Me-cAMP (007) or 10 μM forskolin plus 10 μM rolipram (F/R) for the indicated time points. The percentage change in FRET activity (indicating EPAC activation) is displayed in graphical form. Supplementary Fig. 4. EPAC1-promoted cell spreading occurs independently of AKT activation. A) HEK293T–EPAC1 cells were stimulated for 60 min with either 10 μM Forskolin plus 10 μM Roli [file mmc1.pdf]
